# Marine Floral Biodiversity, Threats, and Conservation in Vietnam: An Updated Review

**DOI:** 10.3390/plants12091862

**Published:** 2023-04-30

**Authors:** Manh-Linh Nguyen, Myung-Sook Kim, Nhu-Thuy Nhat Nguyen, Xuan-Thuy Nguyen, Van-Luong Cao, Xuan-Vy Nguyen, Christophe Vieira

**Affiliations:** 1 Institute of Marine Environment and Resources, VAST, Hai Phong 180000, Vietnam; 2 Faculty of Ecology and Biological Resources, Graduate University of Science and Technology, VAST, Ha Noi 100000, Vietnam; 3 Research Institute for Basic Sciences, Jeju National University, Jeju 63000, Republic of Korea; 4 Institute of Oceanography, VAST, Nha Trang 650000, Vietnam

**Keywords:** algae, Cyanobacteria, Rhodophyta, Ochrophyta, Chlorophyta, conservation, East Vietnam Sea, Biển Đông, molecular-assisted alpha taxonomy, seagrass, seaweed, South China Sea

## Abstract

**Simple Summary:**

The present review provides (1) an updated checklist of the Vietnamese marine flora, (2) a review of molecular-assisted alpha taxonomic efforts, (3) an analysis of marine floral biodiversity spatial distribution nationally and regionally (South China Sea), (4) a discussion on the impact of anthropogenic and environmental stressors on the Vietnamese marine flora, and (5) the efforts developed in the last decade for its conservation. The updated checklist consists of 878 species, including 439 Rhodophyta, 156 Ochrophyta, 196 Chlorophyta, 87 Cyanobacteria, and 15 seagrasses. The South Central Coast supports the highest species diversity of marine algae, which coincides with the largest density of coral reefs along the Vietnam coast. Vietnam holds one of the richest marine floras in the South China Sea owing to the country’s coastline length and associated marine habitat diversity. However, the Vietnamese marine floral biodiversity is facing critical threats, and present management efforts are yet insufficient for their conservation. A methodical molecular-assisted re-examination of Vietnam marine floral biodiversity is urgently needed, complemented with in-depth investigations of the main threats targeted against it; and finally, conservation measures should be urgently implemented.

**Abstract:**

Part of the Indo-Chinese peninsula and located on the northwest edge of the Coral Triangle in the South China Sea, the Vietnamese coastal zone is home to a wealthy marine biodiversity associated with the regional geological setting and history, which supports a large number of marine ecosystems along a subtropical to tropical gradient. The diversity of coastal benthic marine primary producers is also a key biological factor supporting marine biological diversity. The present review provides: (1) an updated checklist of the Vietnamese marine flora, (2) a review of molecular-assisted alpha taxonomic efforts, (3) an analysis of marine floral biodiversity spatial distribution nationally and regionally (South China Sea), (4) a review of the impact of anthropogenic and environmental stressors on the Vietnamese marine flora, and (5) the efforts developed in the last decade for its conservation. Based on the studies conducted since 2013 and the nomenclatural changes that occurred during this period, an updated checklist of benthic marine algae and seagrasses consisted in a new total of 878 species, including 439 Rhodophyta, 156 Ochrophyta, 196 Chlorophyta, 87 Cyanobacteria, and 15 phanerogam seagrasses. This update contains 54 new records and 5 new species of macroalgae. The fairly poor number of new records and new species identified in the last 10 years in a “mega-diverse” country can be largely attributed to the limited efforts in exploring algal biodiversity and the limited use of genetic tools, with only 25.4% (15 species) of these new records and species made based on molecular-assisted alpha taxonomy. The South Central Coast supports the highest species diversity of marine algae, which coincides with the largest density of coral reefs along the Vietnamese coast. Vietnam holds in the South China Sea one of the richest marine floras, imputable to the country’s geographical, geological, and climatic settings. However, Vietnam marine floral biodiversity is under critical threats examined here, and current efforts are insufficient for its conservation. A methodical molecular-assisted re-examination of Vietnam marine floral biodiversity is urgently needed, complemented with in-depth investigations of the main threats targeting marine flora and vulnerable taxa, and finally, conservation measures should be urgently implemented.

## 1. Introduction

Located along the eastern margin of the Indo-Chinese Peninsula, on the northwest edge of the Coral Triangle biodiversity hotspot [1], in the South China Sea (also known as the East Vietnam Sea or Biển Đông), the Vietnamese coastal zone is home to a remarkably rich marine biodiversity [2,3]. Vietnam has been listed among the top 25 most biologically diverse countries in the world [4] and characterized by some authors as a “mega-diverse country” [5,6]. The origin of this diversity is linked to the region’s (Southeast Asia) geological and climatic history, the country’s coastline length covering some 3260 km along a north–south orientation, thus with a wide latitudinal range (stretching from 21°30′ N to 8°25′ N), spanning a subtropical–tropical transition zone, which supports no less than 20 types of marine ecosystems [7]. It is nonetheless important to also point to the role of coastal benthic marine primary producers (e.g., algae, corals, seagrasses) as a key biological factor supporting other forms of marine biological diversity [8]. Benthic marine algae occur across virtually all marine coastal systems from intertidal zone to depths of >200 m, on soft (e.g., sandy) to hard (e.g., rocky) substrates, in a variety of habitats (e.g., lagoons, bays, islands, islets, atolls, and reefs) and ecosystems (e.g., mangroves, seagrasses beds, and coral reefs).

**Figure 1:**
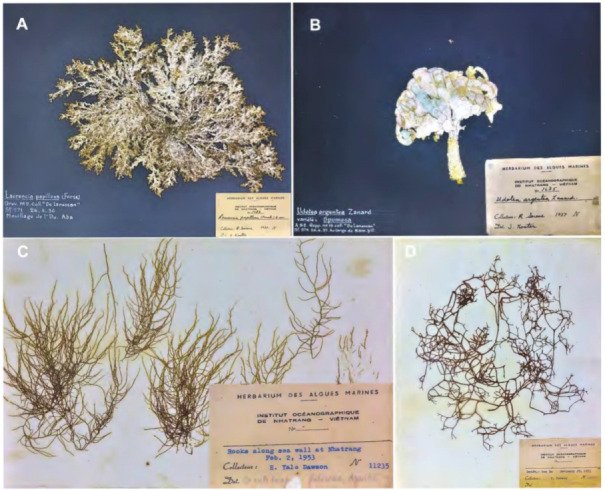
Historical herbarium voucher specimens at Institution of Oceanography—Nha Trang. (**A**) *Laurencia papillosa*, (**B**) *Udotea argentea* collected at Spratly Archipelago from De Lanessan [15], (**C**) *Grateloupia filicina*, and (**D**) *Chnoospora implexa* collected by Dawson [16] in Nha Trang.

The marine flora of Vietnam, which includes three main classes of macroalgae (Chlorophyta, Ochrophyta, Rhodophyta), marine phanerogams or seagrasses (Alismatales), and Cyanobacteria (Cyanophyceae), has attracted the attention of marine botanists since the 1800s. The first mention of Vietnamese seaweeds appeared in the *Flora Cochinchinensis* [9], with the record of 11 names of marine macroalgal species, later referenced in the work of Agardh [10,11] and De Toni [12,13]. The French institution “Institut océanographique de l’Indochine”, corresponding today to the “Institute of Oceanography”, contributed considerably during the 1930s to the knowledge on the algal diversity of Vietnam, notably owing to the work of Pham-Hoàng Hộ [14], considered to be the first Vietnamese marine algal taxonomists of Vietnam. The first collections from the Spratly Islands Archipelago, by the French naturalist Jean Marie Antoine De Lanessan in 1936 (Gouvernement Général de L’Indochine, 1936) [15]—currently housed at the Museum of Oceanography, Nha Trang City—are worthy of mention (Figure 1). Dawson [16] published the very first checklist on the Vietnamese marine flora of Nha Trang Bay in the province of Khanh Hoa (South Central Coast region) with the “Institut Océanographique de Nha Trang”. Dawson [16] reported a total of 204 species (16 Cyanophyceae, 118 Rhodophyta, 22 Ochrophyta, and 48 Chlorophyta), nearly all of which were new species records for Vietnam. Until 1967, the list of marine macroalgae in South Vietnam consisted of 517 species and subspecies [14]. In north of Vietnam, Nguyen et al. [17] provided a list of 281 species. Subsequent studies focused mainly on understudied taxonomic groups and regions of Vietnam, for example, on the families Sargassaceae [18,19,20] and Halymeniaceae [21,22] and on the genera *Gracilaria* and *Gracilariopsis* [23,24], *Eucheuma* and *Kappaphycus* [25,26], *Dictyota* [27], and *Laurencia* [28,29]. Regions of Vietnam that were later investigated included archipelagoes or offshore islands, such as the Spratly Archipelago [30,31,32,33,34], Ly Son Island [35], Phu Quy Island [36], and some inland coastal sites, such as Hai Van–Son Cha [37], Nha Trang Bay [38], Ninh Thuan [39], and Con Dao Island [40]. Recently, Belous et al. [41] published a checklist of 702 species including Rhodophyta, Ochrophyta, and Chlorophyta recorded from Southern and Central Vietnam (16°12′ southward) [41]. Earlier, in 2013, Nguyen et al. (2013) published the first comprehensive marine macroalgal checklist for all Vietnam based on a review of 81 books and publications [42]. The checklist contained a total of 827 species, including 412 Rhodophyta, 180 Chlorophyta, 147 Ochrophyta, and 88 Cyanobacteria. The authors suggested that several taxa needed further investigation to better understand their diversity in Vietnamese waters, and noted the need to combine DNA barcoding and morphological observations to resolve or clarify taxonomic uncertainties. Previously, the checklist of seagrass showed 14 species [43]. In addition, *Halophila major* (Zollinger) Miquel was newly recorded in Vietnamese waters in 2013 [44]. Recently, the phylogenetic analysis inferred from genetic marker and morphological observation revealed the putative hybridization form between two species, *H. ovalis* and *H. major* [45]. No new record or new species of Cyanobacteria were made since the work of Pham-Hoàng Hộ [14].

The aims of the present review were multifold: (1) to deliver an updated checklist of the Vietnamese marine flora, (2) to review molecular-assisted alpha taxonomic efforts implemented in its study, (3) to examine marine floral biodiversity spatial patterns across Vietnamese regions and countries in the South China Sea region, (4) to discuss anthropogenic and environmental threats directly affecting Vietnamese marine flora, and (5) the efforts deployed nationally in the last decade for its conservation.

## 2. Species Diversity Update

Our first objective was to thoroughly review the taxonomic work conducted on the Vietnamese marine flora in the last 10 years since the checklist of Nguyen et al. [42] was published, focusing on 3 main macroalgal phyla/classes (Chlorophyta, Ochrophyta, and Rhodophyta), Cyanobacteria (Cyanophyta), and marine phanerogams or seagrasses (Alismatales). Our literature review of the past 10 years’ research on Vietnamese algae and seagrass taxonomy led to the documentation of a total of 54 new records (i.e., species newly recorded from Vietnam in the period between the last checklist and this updated checklist) and 5 new species (i.e., species newly described from Vietnam in the period between the last checklist and this updated checklist) of macroalgae (3 Rhodophyta, 1 Ochrophyta, and 1 Chlorophyta). New species and new records made during the last 10 years are listed in Table 1. No new records of Cyanobacteria or seagrasses were made since 2013. Based on these new data and the nomenclatural changes that occurred in the last 10 years (such as synonymies), we provide an updated checklist of the benthic marine flora, bringing the new total to 878 species of algae in 42 orders, 108 families, and 284 genera, and consisting of 439 Rhodophyta (21 orders, 52 families, and 161 genera), 156 Ochrophyta (7 orders, 13 families, and 36 genera), 196 Chlorophyta (6 orders, 23 families, and 44 genera), 87 Cyanobacteria (8 orders, 18 families, 42 genera), and 15 seagrasses in Alismatales (1 order, 4 families, and 10 genera) listed in Table 2. A total of 112 species of Rhodophyta are updated to the currently accepted names. Three new species and 13 new records were added to the checklist. Four species, including *Gracilaria mammillaris*, *Dasya baillouviana*, *Meristotheca papulosa*, and *Mesophyllum erubescens*, were removed from the checklist because they were misidentifications. For Chlorophyta, 17 species were updated to the corrected name; 11 species were newly recorded, mainly in 2 genera, *Ulva* and *Caulerpa*; and only 1 new species was new to science. Two species of Chlorophyta were removed from the checklist: the misspelled name (i.e., orthographic mistake in AlgaeBase) “*Codium tunue*” and *Cladophora adhaerens* Ruprecht, which is an invalidly published name. For Ochrophyta, only 1 new species and 2 new records were added to the checklist, and 7 species were updated to the currently accepted names. Finally, 21 species of Cyanobacteria were updated to currently accepted names. We compared our updated checklist with the data available from AlgaeBase [46] for Cyanobacteria, Rhodophyta, Ochrophyta, and Chlorophyta. AlgaeBase [46] data consisted of a lower number of species with a total of 862 species consisting of 51 Cyanobacteria, 438 Rhodophyta, 171 Ochrophyta, and 202 Chlorophyta. Among 4 phyla, Cyanobacteria showed the lowest richness (87 species, 10%). The checklist of marine macroalgae in Vietnam published by Nguyen et al. [42] did not show any new species and new records of Cyanobacteria [42]. The checklist of marine macroalgae in South Vietnam [41], Nha Trang Bay [38], and Con Dao Island [40] did not list any Cyanobacteria. It indicated that Cyanobacteria have not been studied in detail. Therefore, Cyanobacteria need to be included in future works.

## 3. Molecular-Assisted Alpha Taxonomy of the Vietnamese Marine Flora

The use of molecular-assisted alpha taxonomy of marine algae is very recent in Vietnam [24,93]. Molecular tools are presently needed among other purposes: (1) validate previous species identification, (2) identify new records and species, and (3) detect introduced species (e.g., [94,95,96]). Studies combining DNA-based species delimitation techniques and detailed morphological observations have refined our knowledge on Vietnamese species taxonomy and on the individual species’ biogeographical ranges. Nevertheless, such efforts have been very limited in the last decade. Among the 59 new records and species made in the last 10 years, only 25.4% (15 species) were based on molecular-assisted alpha taxonomy. Hereafter, we reviewed molecular studies conducted thus far on Vietnamese marine macroalgae, identifying the taxa studied, marker used, and taxonomic results.

### 3.1. Molecular-Assisted Alpha Taxonomy of Rhodophyta

**Figure 2:**
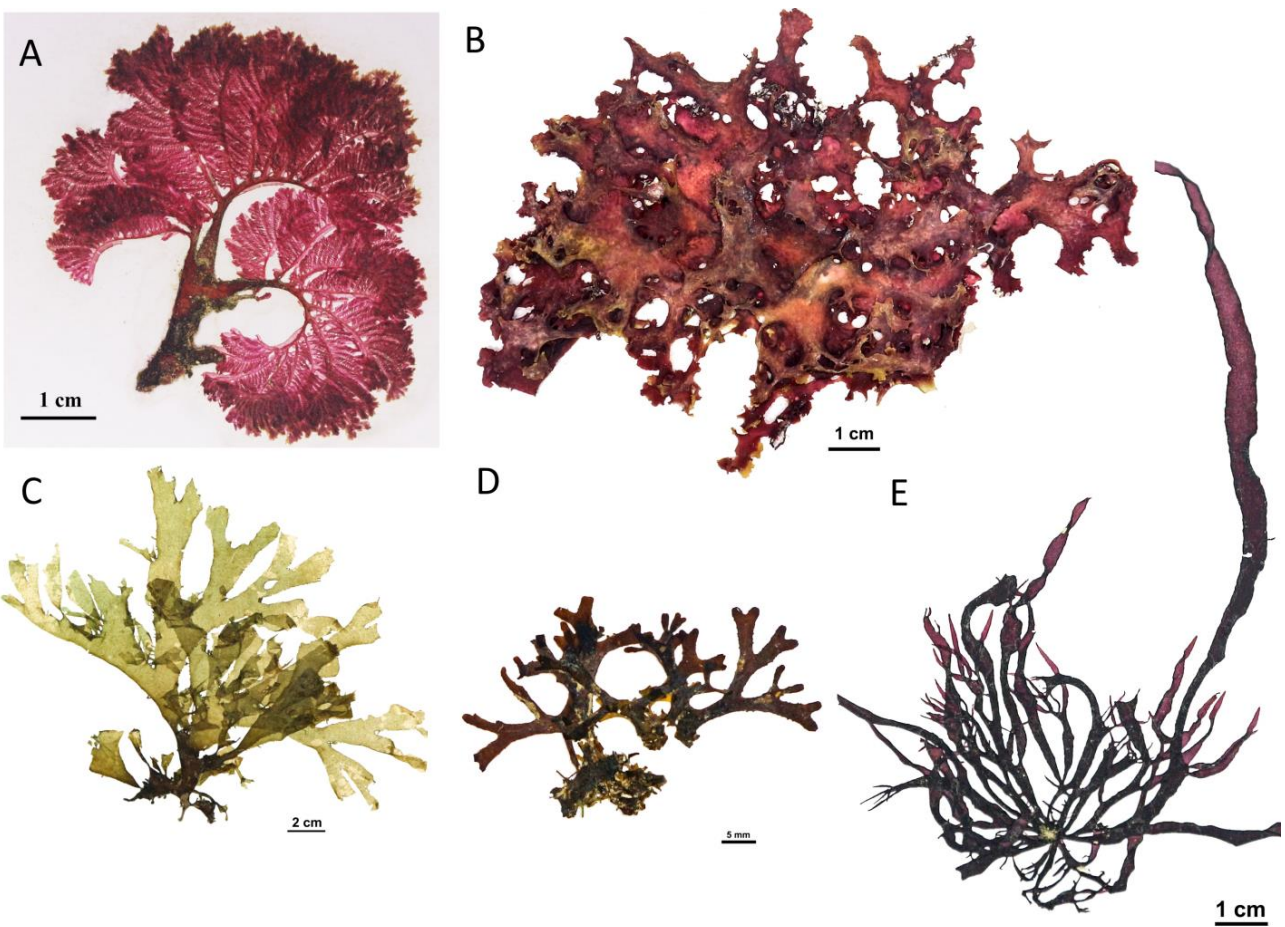
Some recently newly described species and new records made of marine macroalgae from Vietnam. (**A**) *Zellera tawallina* Martens collected at Nha Trang Bay; (**B**) *Meristotheca lysonensis* Nguyen, Nguyen, Kittle & McDermid collected at Ly Son Island; (**C**) *Dictyota hauckiana* Nizamuddin collected at Ninh Thuan; (**D**) *Dictyota grossedentata* De Clerck & Coppejans collected at Nha Trang Bay; (**E**). *Phyllymenia huangiae* (Lin & Liang) Lin, Rodríguez-Prieto, De Clerck & Guiry collected at Da Nang.

Molecular-assisted alpha taxonomic studies on Rhodophyta have comprised a total of four markers, analyzed individually or combined, consisting of two chloroplast genes (large subunit of ribulose-1,5-bisphosphate-carboxylase-oxygenase (*rbc*L); photosystem I P700 chlorophyll a apoprotein A1 (*psa*A)), one mitochondrial gene (cytochrome c oxidase I (*cox*1)), and one nuclear gene (LSU rDNA (28S)). The plastidic *rbc*L gene has been mostly used. The 2006 publication by Hau et al. [24] conducted one of the first molecular studies on Vietnamese Rhodophyta, analyzing the phylogenetic relationships among Gracilariaceae using *rbc*L, which revealed a new species of *Gracilariopsis*, *Gracilariopsis nhatrangensis* Le & Lin. Based on *rbc*L alone, Le et al. [49] later showed that *Gracilaria mammillaris* (Montagne) M.Howe had been misidentified as *Gracilaria phuquocensis* Le, Muangmai & Zuccarello, a new species found in Vietnam; Nguyen et al. [56] newly recorded the Halymeniales species *Phyllymenia taiwanensis* (Lin & Liang) Lin, Rodríguez-Prieto, De Clerck & Guiry in Da Nang from Central Vietnam; Nguyen et al. [55] recorded *Phyllymenia huangiae* (Lin & Liang) Lin, Rodríguez-Prieto, De Clerck & Guiry (Figure 2E); and Duy [47] reported the Rhodomelaceae species *Chondrophycus tronoi* (Ganzon-Fortes) Nam from Vietnam. Analyses based on *cox*1, *psa*A, and *rbc*L sequences allowed the discovery of the Gelidiellaceae species *Perronella gracilis* Boo, Nguyen, Kim & Boo from Nha Trang Bay from Southern Vietnam [53], and the transfer of *Gelidiella adnata* Dawson to *Parviphycus adnatus* (Dawson) Santelices. Analyses based on the concatenated *rbc*L and *cox*1 sequences also revealed a new record of Delesseriaceae from Vietnam, *Zellera tawallina* Martens (Figure 2A), previously identified as *Claudea batanensis* Tanaka [55]. Analyses combining *rbc*L and *cox*1 sequences allowed the identification of a new species, *Meristotheca lysonensis* Nguyen, Nguyen, Kittle & McDermid, collected at Ly Son Island in the South Central Coast region of Vietnam [52] (Figure 2B). A last worthy account for Rhodophyta is that of the Halymeniaceae species *Halymenia dilatata* Zanardini, a common species in Vietnam, previously reported in several publications [14,16]. Based on phylogenetic analyses using concatenated chloroplast and mitochondrial and nuclear markers (*rbc*L, *cox*1, and LSU rDNA (28S)), Vy et al. [50] showed that *H. dilatata* may have been misidentified as *Halymenia malaysiana* Tan, Lim, Lin & Phang, a study that confirms new distributional records of *Phycocalidia tanegashimensis* along the Chinese and Vietnamese coastline in the South China Sea. The study used molecular sequence data from rbcL, COI-5P, and 18S rRNA genes to place *P. tanegashimensis* in a clade with *P. acanthophora*, *P. denticulata*, *P. suborbiculata*, and *P. vietnamensis* as out-groups [54].

### 3.2. Molecular-Assisted Alpha Taxonomy of Ochrophyta

Molecular-assisted alpha taxonomic studies on Ochrophyta have comprised a total of four molecular markers, analyzed individually or combined, consisting of two chloroplast genes (*rbc*L and the PSII thylakoid protein D1 (*psb*A)), one mitochondrial gene (cytochrome c oxidase subunit III (*cox*3)), and one nuclear encoded ribosomal cistron (ITS 2 rDNA). Tu [97] used ITS2 rDNA and *cox*3 sequences to reassess *Sargassum* species diversity from Vietnam. The order Dictyotales has received particular attention in recent years. Using *rbc*L and *psb*A markers, Nguyen-Nhat et al. [58] newly identified *Dictyota hauckiana* Nizamuddin from Ninh Thuan (Figure 2C). One additional species of *Dictyota* was newly recorded from Vietnam, *Dictyota grossedentata* De Clerck & Coppejans [55] (Figure 2D). Molecular phylogenetic analyses based on concatenated *rbc*L and *cox*3 sequences led to the description of the new species *Lobophora tsengii* Tien & Sun from Bach Long Vy [60], although morphological and molecular analyses did not conclusively rule out its conspecificity with *Lobophora rosacea* C.W.Vieira, Payri& De Clerck.

### 3.3. Molecular-Assisted Alpha Taxonomy of Chlorophyta

For Chlorophyta, the only molecular-assisted alpha taxonomic study reported until now is that by Tran et al. [61], who reassessed the species diversity in Vietnam of the Ulvaceae genus *Ulva* based on *rbc*L and the elongation factor Tu (*tuf*A). The study revealed seven new records of *Ulva* from Vietnam and identified a new species, *U. vietnamensis* L-A. T. Tran, Leliaert & De Clerck.

### 3.4. Molecular-Assisted Alpha Taxonomy of Cyanobacteria and Alismatales

For Alismatales (seagrasses), the concatenated rbc*L* and mat*K* were applied to assess the species diversity of *Halophila* [98]. Based on the genetic marker ITS, a later study by Nguyen et al. [44] showed that *Halophila major* was the correct name for the collections of *Halophila ovalis* from Nha Trang Bay. All seagrass species from Vietnam were confirmed with molecular markers, and samples previously labeled as “*Halophila johnsonii*” were reidentified as *H. ovalis.* Therefore, *Halophila johnsonii* was removed from the seagrass checklist of Vietnam [99]. *Halophila major* was found in most offshore islands, whereas *H. ovalis* occurred in lagoons in Vietnamese waters [100]. In contrast, no molecular-assisted alpha taxonomic study on Cyanophyceae was yet conducted in Vietnam.

### 3.5. Intraspecific Genetic Diversity Studies

**Figure 3:**
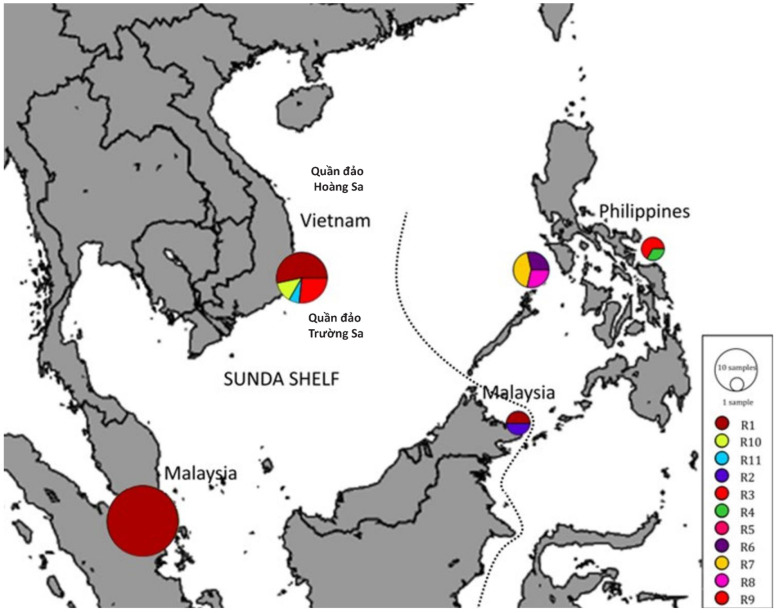
Distribution of haplotypes of *Halymenia malaysiana* in Sunda Shelf (Malaysia and Vietnam) and the Philippines [50]. Adapted from Nguyen et al. [50].

Several DNA fingerprinting have been applied to investigate the genetic relationships among individuals within or among populations of the same species [101,102]. In a global study of *Gracilaria salicornia* (Agardh) Dawson from Southeast Asia, Yang et al. [103] distinguished a lineage of the Philippines from other Southeast Asian countries (e.g., Malaysia and Thailand). For another Rhodophyta, *Phycocalidia acanthophora* (Oliveira & Coll) Santiañez, the dataset of *rbc*L indicated that there is no haplotype sharing between populations in the Philippines and other nearby areas, including Taiwan, Japan, and Hong Kong [104]. In Vietnam, the red algae species in *Kappaphycus* and *Eucheuma* are important economically and were widely cultivated in the South Central. So far, based on a combined *cox*2–3 and *rbc*L dataset, Zuccarelo et al. [105] compared the genetic variation among cultivated *Kappaphycus alvarezii* (Doty) Liao farming worldwide, including a strain from Vietnam; the authors indicated that there is no genetic variation among samples collected in Vietnam and other Southeast Asian countries, such as the Philippines, Malaysia, and Indonesia. However, *Kappaphycus alvarezii* collected from Africa and Hawai’i showed significant differences from populations in Southeast Asian countries. By using random amplified polymorphic DNA (RAPD) markers, Hong et al. [106] also revealed the genetic variation among strains of *Kappaphycus* spp. and *Eucheuma* spp. in Vietnam, *Kappaphycus striatus* (Schmitz) Liao. The analyses of the mitochondrial *cox*2–3 spacer of *Kappaphycus* spp. and *Eucheuma* spp. showed that there are two haplotypes of *K. alvarezii*, and an unidentified *Kappaphycus* sp. was also found in Vietnam and the Philippines [107]. A later study by Tan et al. [108] indicated that the aring-aring strain was described as the new species *Kappaphycus malesianus* Tan, Lim & Phang. There is no evidence of occurrence of this species in Vietnamese waters. The biogeography of *Halymenia malaysiana* was studied in more detail. Our previous study showed that the common haplotype in Vietnam is R1, and three new haplotypes were added to *H. malaysiana* for Southeast Asia (Figure 3). There are statistically significant genetic differences between Sunda Shelf (Vietnam and Malaysia) populations and those in Philippine waters [50]. For another economic species, *Gracilaria tenuistipitata* Chang & Xia, Song et al. [109] found that there is only one haplotype (T5) in Vietnam. Compared with other haplotypes in Thailand, Malaysia, and Singapore, a haplotype of *Gracilaria tenuistipitata* collected in Vietnam showed from one to eight mutational steps. Recently, the *tuf*A gene was applied to find the haplotype and genetic diversity of the green algae *Halimeda* spp.; Nguyen et al. [110] concluded that the genetic variation in *H. macroloba* Decaisne is very low, and *H. opuntia* (Linnaeus) J.V.Lamouroux tends to form a distinct group in Vietnamese waters.

## 4. Biodiversity Distribution Patterns

### 4.1. Marine Floral Biodiversity across the South China Sea

**Figure 4:**
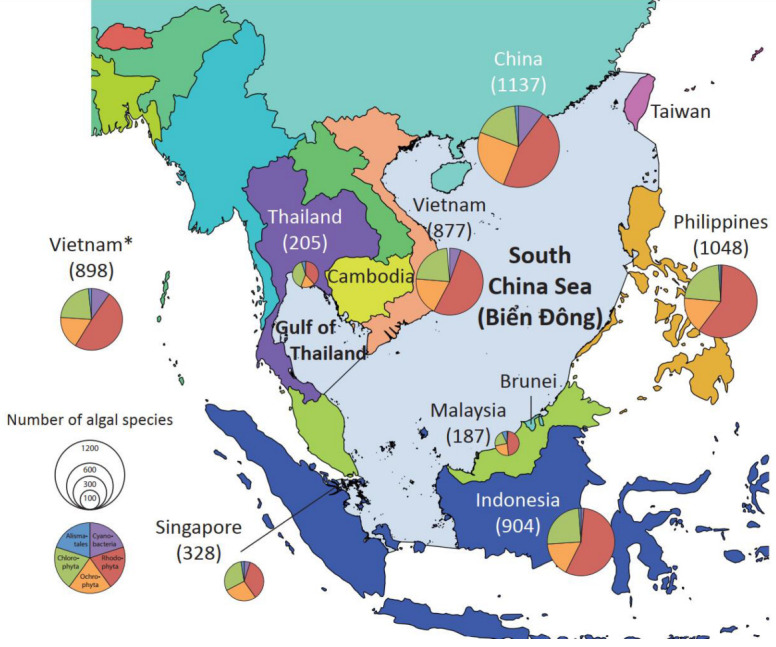
Spatial variation in marine floral biodiversity (Cyanobacteria, Rhodophyta, Ochrophyta, and Chlorophyta) across countries bordering the South China Sea (East Vietnam Sea). Marine floral biodiversity data based on AlgaeBase [46]. * Data for the Vietnam marine flora based on the current updated checklist. Colors mean blue is Alismatales, green is Chlorophyta, red is Rhodophyta, brown is Ochrophyta, and purple is Cyanobacteria.

Located on the northwest edge of the Coral Triangle biodiversity hotspot, the South China Sea is one of the most productive marine regions in the world [111,112]. The sea is bordered by twelve states and territories, including Brunei, Cambodia, (mainland) China, Hong Kong, Indonesia, Macao, Malaysia, the Philippines, Singapore, Taiwan, Thailand, and Vietnam. Phang et al. [63] documented 1412 species of marine algae from the South China Sea (119 Cyanophyceae, 305 Chlorophyta, 258 Ochrophyta, and 730 Rhodophyta) from six countries bordering the South China Sea (Indonesia, Malaysia, the Philippines, Singapore, Thailand, and Vietnam). Their analyses showed similarity in the marine algal floras of Malaysia, Singapore, and Thailand and those of Vietnam and the Philippines. We present here an overview of species diversity from states and territories bordering the South China Sea based on AlgaeBase [46] for four algae classes (Cyanobacteria, Rhodophyta, Ochrophyta, and Chlorophyta) and seagrasses. No data were available on AlgaeBase for Brunei, Hong Kong, Macau, Cambodia, and Taiwan. Data for seagrasses were retrieved from different sources indicated in Figure 4 and Table 3. The Gulfs of Thailand and Tonkin were included in the South China Sea. It should be noted that with the exception of Vietnam, the species numbers provided here are not restricted to the South China Sea, but are all-inclusive for each country (i.e., not restricted to the South China Sea), and retrieving data restricted to the South China Sea was not possible. In comparison with other South China Sea bordering states/territories, Vietnam supports the fourth highest marine floral diversity with 877 species, according to AlgaeBase [46] (but 881 species according to our updated checklist). However, taking into account the all-inclusiveness of the number for other countries, Vietnam possibly holds the highest diversity in the South China Sea. In fact, the South China Sea coastlines of the three other species-rich states (China, the Philippines, Indonesia) represent only a fraction of these countries.

Biodiverisity numbers should nevertheless be interpreted cautiously as they may under-represent the actual floral diversity of each country and the region, since they are for the most part established on morphological-based identification, and additionally, some countries have received much lesser attention than others (e.g., Brunei, Cambodia, and Malaysia). Notwithstanding, the high floral biodiversity in the South China Sea documented so far from Vietnam can be attributed to its geographical location, situated along the southeastern margin of the Indo-Chinese Peninsula, comprising the largest area of the peninsula and the longest coastline in the South China Sea.

**Figure 5:**
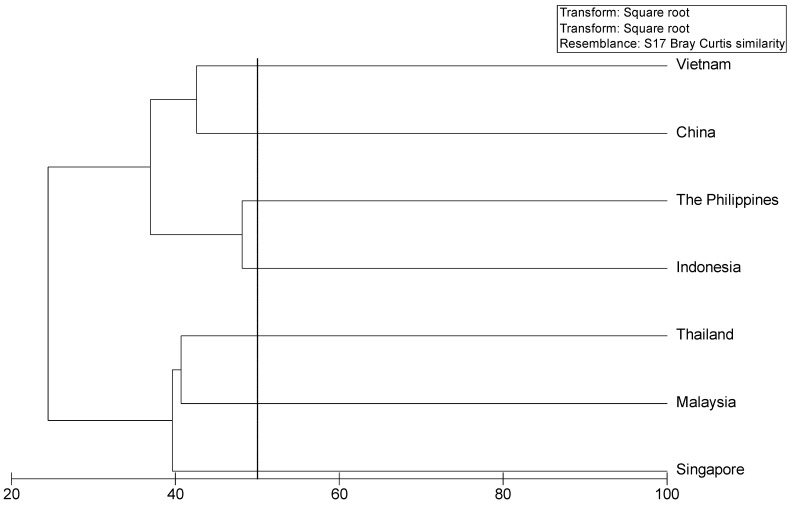
Cluster analyses of similarity (Bray–Curtis index) for the marine floras of the countries bordering the South China Sea. Data for similarity analyses range between 20 and 100. Hierarchical clustering is based on square-root-transformed presence data and on a resemblance matrix calculated using S17 Bray–Curtis similarity.

We examined the similarity of the marine floras in seven of the states and territories bordering the South China Sea using a Bray–Curtis similarity index [120] multivariate analysis implemented in Primer V.6 software [121] based on compiled data for the region. Results showed that the Vietnamese marine flora was most similar to that of China, followed by those of Indonesia and the Philippines (Figure 5), and that the marine floras of Malaysia, Singapore, and Thailand were very similar (Figure 5), consistent with previous findings by Phang et al. [63].

### 4.2. Marine Floral Biodiversity across Vietnam Regions

**Figure 6:**
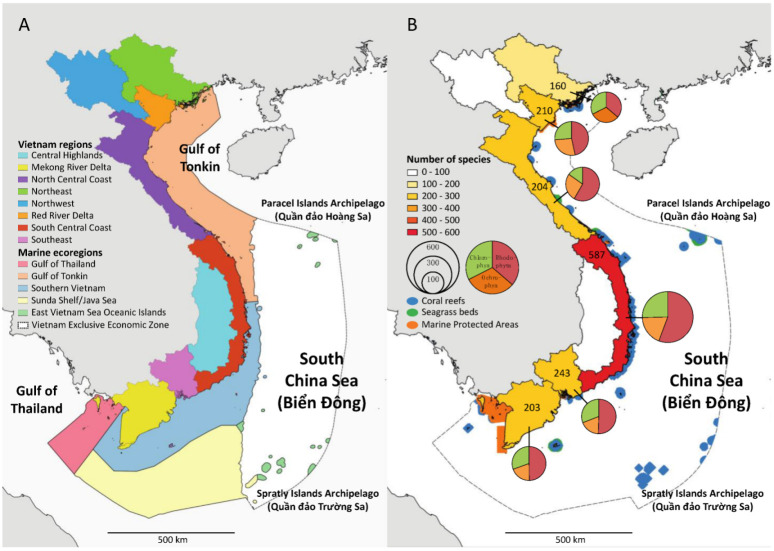
Spatial variation in marine floral biodiversity across Vietnamese regions. (**A**) Illustration of the Vietnamese administrative regions and the marine ecoregions sensu Spalding et al. [122] within the Vietnamese Exclusive Economic Zone (VEEZ). (**B**) Representation of species diversity across marine macroalgal classes (Cyanobacteria, Rhodophyta, Ochrophyta, and Chlorophyta) and depiction of coral reefs, seagrass beds, and marine protected area distributions within the VEEZ; numbers in each region represent the total number of species.

The Vietnamese coastline is divided into three “Geographical Regions” (Northern Vietnam, Central Vietnam, Southern Vietnam) subdivided into eight “Administrative Regions” (Northeast, Northwest, Red River Delta, North Central Coast, South Central Coast, Central Highlands, Southeast, Mekong River Delta) (Figure 6A). Based on our updated checklist, we show marine floral biodiversity across the “Administrative Regions”, excluding the Northwest and Central Highlands regions, which have no coastline. The geographical distribution of the marine floral biodiversity is uneven across Vietnamese regions. The South Central Coast holds the highest diversity by far, with a total of 587 species, followed by the Southeast (243), Red River Delta (210), North Central Coast (204), and Mekong River Delta (203) regions. The Northeast region is the least species region with 160 species. The high marine floral biodiversity documented in the South Central Coast coincides with the largest coral reef density along the coastline of Vietnam (Figure 6B) and a high diversity of marine environments [3]. It is worth mentioning that Spalding [122] proposed five marine ecoregions (Gulf of Thailand, Gulf of Tonkin, Southern Vietnam, Sunda Shelf/Java Sea, South China Sea (East Vietnam Sea) Ocean Islands) along the Vietnamese coastline (Figure 6A).

According to present data, with only 45 endemic species, Vietnam would seem to contain a very low level of endemism of marine flora (5.01%, 45 spp.; Table 4). However, this number most likely under-represents the actual level of endemism for the marine flora of Vietnam, since molecular-assisted alpha taxonomic efforts, needed to obtain accurate taxonomic data, have been very limited.

### 4.3. Seaweed Biodiversity Loss, Threats, and Conservation

Some species of marine algae in Vietnam have experienced declines in their populations due to a variety of factors. Several threats to marine algae exist in Vietnam, including but not restricted to pollution, climate change, overharvesting, invasive species, and habitat destruction. Marine algae are vulnerable to pollution from a variety of sources, including agricultural runoff, industrial discharge, and sewage. Pollution can harm marine algae directly and also make their habitat less suitable for growth [123]. Marine algae are sensitive to changes in temperature, salinity, and other environmental conditions, and they may be negatively impacted by climate change [124]. Some species of marine algae are in high demand for use in food, cosmetics, and other products, and overharvesting can lead to a decline in their populations [125]. Non-native species of marine algae that are introduced to new areas can outcompete native species and reduce their populations [126]. Marine algae rely on specific types of habitat for growth, and the destruction of these habitats can negatively impact their populations [127]. The biodiversity (marine and terrestrial) of Vietnam has decreased quickly [128]. Some of the known factors in Vietnam are land conversion without a proper scientific base, quick reduction of natural forests, infrastructure developments (e.g., dams, roads, and new urban and rural human settlements), and overexploitation of natural resource/illegal exploitations in fishing, hunting, forestry [2,129,130]. It is difficult to quantify the extent of marine algal diversity loss in Vietnam, as there are limited data available on this topic. Titlyanov et al. [38] quantified seaweed community changes in Nha Trang Bay and investigated the factors associated with these changes. Collections sampled between 1953 and 1968 and 1982 and 1987 did not change significantly in either the species diversity nor the floristic composition. However, the species composition assessed between 2002 and 2010 showed changes in the species diversity composition, with an increase inf Chlorophyta and a reduction of Rhodophyta and Ochrophyta species. In Con Dao Island, significant changes in marine floral species composition were observed between 1998 and 2008, with a proportional species replacement in each taxonomic group over the last two decades [40]. Since the 1970s, several species have not been observed, such as *Erythrocladia irregularis* Rosenvinge, *Acrochaetium crassipes* (Børgesen) Børgesen, *Metagoniolithon stelliferum* (Lamarck) Ducker, and *Exophyllum wentii* Weber Bosse [41]. Similarly, the species diversity of *Sargassum* was previously well studied at Nha Trang Bay, with the identification of 21 species between 1950 and 1970. Between 1980 and 2000, 9 of the previously identified species were not recorded, while an additional 15 species were newly added to *Sargassum*. However, based on the most recent collection, in 2020 in Nha Trang Bay, *Sargassum* was represented by 14 species, including 7 species found in the previous two surverys and 7 new additions. Overall, 24% (149 species) of algal species in South Vietnam recorded between 1980 and 2000 could not be found between 2000 and 2020 [41]. A report by Vy et al. [131] indicated that nearly 50% of the *Sargassum* beds at Hon Chong (Khanh Hoa Province) have disappeared because of loss of substratum, and the species *Sargassum crassifolium*, once a dominant species in this site, disappeared. In Nha Trang Bay, seawater pollution resulting from dissolved organic and inorganic compounds of nitrogen and phosphorus may lead to an increasing larger number of green algae and their biomass as well as population density. The green algae may displace fleshy and foliose forms of red and brown macroalgae from communities [38]. Another threat to seaweed biodiversity is harvesting of natural stocks. Local harvesters collect large quantities of *Sargassum* for production of alginates, Asian herbal medicine, and various human foods [132]. Young populations of *Sargassum* are commonly harvested prior to reaching sexual maturity and reproduction, thus affecting natural stock renewal (authors’ pers. obs.). Another case of overexploitation of natural stocks was reported in the edible red seaweed *Betaphycus gelatinus*, now very rare due to harvesting by locals at Ninh Thuan Province. The Vietnam Red data Book [133] shows 8 and 5 species of Rhodophyta and Ochrophyta, respectively. Among them, *Crytonemia undulata* is in the critically endangered category. Six species including 5 Rhodophyta and 3 Ochrophyta are in the endangered category. The 6 remaining species are in the vulnerable category. There are 12 marine protected areas (MPAs) from 10 provinces/cites in Vietnam. Large seaweed beds in Khanh Hoa, Ninh Thuan, are out of the core zone of MPAs and therefore under threat. The natural stock of *Hydropuntia eucheumatoides*, *Betaphycus gelatinus*, and *Sargassum* spp. (endangered category) is still collected by local people due to lack of Red Data Book. Like seagrasses, the management models of marine macroalgal ecosystems in Vietnam are mostly integrated into coastal management models to solve the problems of weaknesses that exist in the management, exploitation, and use of natural resources and environmental protection in coastal areas.

## 5. Conservation Efforts

Conservation efforts are needed to protect and conserve marine floral diversity in Vietnam. It is important to address these threats in order to maintain the health and resilience of Vietnam marine environments and the economic and cultural value of these resources. This may involve measures such as habitat conservation, sustainable harvesting, pollution reduction, and invasive species management. There are several conservation efforts underway in Vietnam to protect the marine flora and the marine environments they are a part of. Some of these efforts are reviewed below.

### 5.1. Habitat Conservation

Many conservation efforts in Vietnam focus on protecting and preserving the habitats that support marine flora. This may involve establishing marine protected areas (MPAs) or other types of conservation zones, which are designated areas of the ocean that are set aside for the protection and conservation of marine life. Recognizing the importance of marine protected areas in the protection of marine biodiversity, the prime minister released Decision No. 742/QD-TTg on 26 May 2010, authorizing the preparation for the marine biodiversity scheme. Marine conservation in Vietnam will continue until 2020 (this deadline has been extended), with the aim of preserving habitats and marine species of economic and scientific importance. It aimed to contribute to the development of marine economy and improve the livelihoods of fishermen communities in coastal localities. The Fisheries Law of 2017 was passed by the 14th National Assembly, which includes provisions for the protection and growth of aquatic resources, including marine conservation, in the sense of sustainable fisheries development and international integration. The Communist Party of Vietnam’s Central Committee released Resolution No. 36-NQ/TW on the Strategy for Sustainable Development of Vietnam’s Marine Economy to 2030 with a Vision to 2045 on 22 October 2018. The document stated on the matter: “Sustainable development of the marine economy on the basis of green growth, biodiversity protection, and marine environment conservation; ensure harmony between economic and natural environments, conservation and development, promoting the sea’s potentials and advantages, and creating a driving force for national economic development”; and that the specific target was to “Well maintain and protect aquatic, coastal, and island ecosystems; raise the area of marine and coastal protected areas to at least 6% of the national marine area”. Currently, the Ministry of Agriculture and Rural Development and other cities have created and operationalized 12 marine protected areas (Figure 4B, Table 5). These 12 MPAs amount to a total of 243,023 ha (ca. 2430 km^2^), which corresponds to ca. 0.17% of the total surface (ca. 1,395,096 km^2^) of the Vietnamese Marine Exclusive Economic Zone. Among the 12 MPAs, 3 typically contain seaweeds and 9 seagrasses.

Moreover, there are other nature reserves along the coast of Vietnam [134] (Table 6). The Ministry of Agriculture and Rural Development has developed comprehensive plans for the establishment of four MPAs, which have been submitted to provincial people’s committees for approval: Hon Me/Thanh Hoa, Nam Yet/Khanh Hoa, Phu Quy/Binh Thuan, and Hai Van–Son Cha/Da Nang–Hue.

Despite the fact that the Ministry of Agriculture and Rural Development has organized a mission to inspect, guide, and have several documents to direct and inform, the provincial people’s committees have not yet approved the establishment after more than 5 years of handover of Vietnam’s coastal area has high biodiversity: 13 out of 28 national parks, 22 out of 55 nature reserves, and 17 out of 34 forests of cultural, historical, and environmental significance are located in coastal areas and islands.

### 5.2. Sustainable Harvesting

Some species of seaweed in Vietnam are harvested for use in food, cosmetics, and other products. In order to ensure the sustainable use of these resources, there are efforts to establish sustainable harvesting practices and to manage fisheries to ensure that seaweed populations are not overharvested. Vietnam adopted international standards of the sanitary and phytosanitary (SPS) agreement–based regulation, which includes seaweeds. This established regulation covers a wide range of standards, including ensuring that the seaweeds are disease-, pathogen-, toxin-free, and furthermore that seaweeds meet permissible levels for heavy metals and other contaminants (e.g., pesticides). Vietnam has national regulations for controlling the movement of aquatic aquaculture organisms (quarantine), which also includes the import of live seaweed. For example, Vietnam provided a technical guideline for importing live seaweed, e.g., *Gracilaria* species [135], and technical requirements for *Kappaphycus alvarezii* (Table 7). 

### 5.3. Pollution Reduction

Seaweeds are vulnerable to pollution from a variety of sources, and efforts are being made to reduce pollution in Vietnam’s coastal waters in order to protect these ecosystems. This may involve measures such as improving wastewater treatment, regulating industrial discharge, and reducing agricultural runoff. However, no particular reports and regulations were found on pollution reduction in Vietnam’s coastal waters.

### 5.4. Invasive Species Management

Non-native species of seaweed that are introduced to new areas can outcompete native species and reduce their populations. To address this threat, efforts are being made to control the spread of invasive seaweed species in Vietnam. Circular No. 35/2018/TT-BTNMT dated 28 December 2018 of MONRE stipulates the criteria for the identification and promulgation of a list of invasive alien species. However, the subject only focuses on species that have been announced under the guidance of Circular 35, and the assessed ecosystems are only terrestrial and aquatic. There is almost no information about groups of marine organisms, including seaweed and seagrasses, more specifically, foreign species that are invasive in the sea and by shipping route; there has not been a specific study in Vietnam. In order to prevent the entry of alien organisms in the ballast water environment transported by ships from other sea areas, affecting the ecosystem, economy, and human health and strengthening measures to protect the marine environment, IMO ratified the BWM Convention on 13 February 2004, and the convention met the conditions to enter into force on 8 September 2017. By 8 September 2024, all ships are required to use a ballast water management system (D2). Vietnam is in the process of completing the procedures to join the convention. The basic legal documents related to the activities of dumping garbage and discharging wastewater and ballast water are specified in Article 117 of Decree 58/2017/ND-CP guiding the Vietnam Maritime Code on the management of cargo operations [136].

## 6. Conclusions: Challenges and Future Directions

Studies conducted in the last decade effectively illustrated the need to combine molecular tools with morphological observations (i.e., habit view, vegetative and reproductive morphology) in (1) the reassessment of marine floral species diversity, (2) previous species names’ validation, (3) misidentification detection, and (4) new species discovery. However, the fairly poor number (15 taxa; 25.4% of the new records and species) of new records and species made over the last 10 years in a “mega-diverse” country raised worrying concerns on the efforts put into the study of marine floral biodiversity. Past molecular-assisted taxonomic efforts have been focused on a limited number of taxa and localities. Currently, three main institutions, including (1) the Institute of Marine Environment and Resources in the North, (2) the Institute of Oceanography in the Central, and (3) the Institute of Tropical Biology in the South, are conducting most studies on marine algae taxonomy nationally. Considering the important length of the Vietnamese coastline (>3200 km), an exhaustive exploration of the Vietnamese marine flora represents a Herculean task for these institutions alone. In addition, the limited number of algal taxonomists in Vietnam and limited funding availability represent a major challenge to the study of marine floral biodiversity. Methodical molecular-assisted re-examination of Vietnam marine floral biodiversity is urgently needed in order to get an accurate picture of biodiversity and endemism, and thereby obtain baseline data for the marine floral management and protection. In particular, future efforts will need to be directed towards specific taxa and regions of Vietnam. Data provided in this review on species diversity, groups targeted with molecular-assisted alpha taxonomic approaches, and spatial variation in biodiversity offer valuable data to orientate future efforts. Finally, a more in-depth investigation of the threats targeting the marine flora of Vietnam is needed, and urgent implementation of measures for its conservation is called for, in particular, the increase in marine protected areas across Vietnam, which represent now less than 1% of the Vietnamese Marine Exclusive Economic Zone.

## Figures and Tables

**Table 1:** List of new species (^a^) and new records (^b^) of marine macroalgae found in Vietnam with support from genetic markers and/or morphological observation since 2013. -/-, as above, na: not available. Ref.: reference.

No.	Phylum	Species	Previous identification in Vietnam	Applied Genetic Markers	Ref.
1 ^b^	Rhodophyta	*Amphiroa crassa* J.V.Lamouroux		na	[41]
2 ^b^		*Antithamnion antillanum* Børgesen		na	-/-
3 ^b^		*Antithamnionella breviramosa* (E.Y.Dawson) Wollaston		na	-/-
5 ^b^		*Caulacanthus ustulatus* (Turner) Kützing		na	-/-
4 ^b^		*Ceramium borneense* Weber Bosse		na	[38]
6 ^b^		*Chondrophycus tronoi* (Ganzon-Fortes) Nam		*rbc*L	[47]
8 ^b^		*Coelothrix irregularis* (Harvey) Børgesen		-/-	[48]
7 ^b^		*Endosiphonia horrida* (C.Agardh) P.C.Silva		na	[41]
9 ^b^		*Gelidium crinale* var. *perpusillum* Piccone & Grunow		na	[38]
10 ^b^		*Gelidium pusillum* var. *cylindricum* W.R.Taylor		na	[41]
11 ^b^		*Gelidium pusillum* var. *pacificum* W.R.Taylor		na	[38]
12 ^b^		*Gloiocladia japonica* (Okamura) Yoshida		na	[41]
14 ^a^		*Gracilaria phuquocensis* Le, Muangmai & Zuccarello	*Gracilaria mammillaris* (Montagne) M. Howe	*rbc*L	[49]
13 ^b^		*Gracilaria divergens* (C.Agardh) J.Agardh		na	-/-
15 ^b^		*Halymenia malaysiana* Tan, Lim, Lin & Phang		*rbc*L, COI-5P, 28S	[50]
16 ^b^		*Hypoglossum simulans* M.J.Wynne, I.R.Price & D.L.Ballantine		na	[38]
17 ^b^		*Laurencia natalensis* Kylin			[48]
19 ^b^		*Melanothamnus thailandicus* (N.Muangmai & C.Kaewsuralikhit) Díaz-Tapia & Maggs		*rbc*L	[51]
18 ^a^		*Meristotheca lysonensis* Nguyen, Nguyen, Kittle & McDermid		*rbc*L, COI-5P	[52]
20 ^b^		*Parviphycus adnatus* (Dawson) Santelices	*Gelidiella adnata* Dawson	*cox*1, *psa*A, *rbc*L	[53]
21 ^a^		*Perronella gracilis* Boo, Nguyen, Kim & Boo		*cox*1, *psa*A, *rbc*L	-/-
24 ^b^		*Phycocalidia tanegashimensis* (I.Shinmura) Santiañez		*rbc*L, COI-5P, 18S	[54]
22 ^b^		*Phyllymenia huangiae* (Lin & Liang) Lin, Rodríguez-Prieto, De Clerck & Guiry		*rbc*L, COI-5P	[55]
23 ^b^		*Phyllymenia taiwanensis* (Lin & Liang) Lin, Rodríguez-Prieto, De Clerck & Guiry		*rbc*L, COI-5P	[56]
25 ^b^		*Plocamium ovicorne* Okamura		na	[41]
26 ^b^		*Pterocladiella bartlettii* (Taylor) Santelices		COI-5P, *cob*, *psa*A, *psbA*, *rbcL*	[57]
27 ^b^		*Pterocladiella maribagoensis* G.H.Boo & P.J.L.Geraldino		-/-	-/-
28 ^b^		*Pterocladiella musciformis* (W.R.Taylor) G.H.Boo & K.A.Miller		-/-	-/-
29 ^b^		*Titanophora pikeana (*Dickie) Feldmann		-/-	[48]
30 ^b^		*Tylotus lichenoides* Okamura		-/-	[41]
31 ^b^		*Zellera tawallina* Martens		*rbc*L, COI-5P	[55]
33 ^b^	Ochrophyta	*Dictyota grossedentata* De Clerck & Coppejans		*rbc*L, *psb*A	[55]
34 ^b^		*Dictyota hauckiana* Nizamuddin		*rbc*L, *psb*A	[58]
32 ^b^		*Dictyota dichotoma* var. *intricata* (C.Agardh) Greville		na	[38]
35 ^b^		*Ectocarpus siliculosus* (Dillwyn) Lyngbye		na	[41]
38 ^b^		*Lobophora obscura* (Dickie) C.W.Vieira, De Clerck & Payri		na	[59]
36 ^b^		*Lobophora papenfussii* (W.R.Taylor) Farghaly		-/-	[47]
37 ^a^		*Lobophora tsengii* Tien & Sun		*rbc*L, *cox*3	[60]
39 ^b^		*Padina japonica Yamada*		na	[41]
40 ^b^		*Turbinaria turbinata* (Linnaeus) Kuntze		-/-	[41]
41 ^b^	Chlorophyta	*Boodleopsis pusilla* (Collins) W.R.Taylor, A.B.Joly & Bernatowicz		-/-	[38]
42 ^b^		*Caulerpa andamanensis* (Taylor) Draisma, Prudhomme & Sauvage		-/-	[48]
		*Caulerpa chemnitzia* var. *laetevirens* (Montagne) Fernández-García & Riosmena-Rodriguez		-/-	[41]
43 ^b^		*Caulerpa calcifolia* Harvey & Bailey		-/-	[48]
44 ^b^		*Caulerpa sertularioides f. longiseta* (Bory) Svedelius		-/-	[41]
45 ^b^		*Caulerpa oligophylla* Montagne		-/-	[48]
46 ^b^		*Cladophora gracilis* Kützing		-/-	[41]
47 ^b^		*Codium intricatum* Okamura		-/-	[38]
48 ^b^		*Chaetomorpha basiretrorsa* Setchell		-/-	-/-
49 ^b^		*Parvocaulis exiguus* (Solms-Laubach) S.Berger, Fettweiss, Gleissberg, Liddle, U.Richter, Sawitzky & Zuccarello		-/-	-/-
50 ^a^		*Ulva vietnamensis* L.-A.T.Tran, Leliaert & De Clerck		ITS, *rbc*L, *tuf*A	[61]
51 ^b^		*Ulva aragoënsis* (Bliding) Maggs		-/-	-/-
52 ^b^		*Ulva chaugulii* Kavale & Kazi		-/-	-/-
53 ^b^		*Ulva kraftiorum* Huisman		-/-	-/-
54 ^b^		*Ulva limnetica* Ichihara & Shimada		-/-	-/-
55 ^b^		*Ulva meridionalis* Horimoto & Shimada		-/-	-/-
56 ^b^		*Ulva ohnoi* Hiraoka & Shimada		-/-	-/-
57 ^b^		*Ulva tepida* Masakiyo & Shimada		-/-	-/-
58 ^b^		*Ulvella leptochaete* (Huber) R.Nielsen, C.J.O’Kelly & B.Wysor		na	[38]
59 ^b^		*Ulvella scutata* (Reinke) R.Nielsen, C.J.O’Kelly & B.Wysor		-/-	/-/

**Table 2:** Checklist of the marine flora (Alismatales, Chlorophyta, Cyanobacteria, Ochrophyta, Rhodophyta) of Vietnam. The checklist is systematically and alphabetically arranged at the phylum, ordinal, familial, generic, and species levels.

Species Name	Regionally Reported as	References
**CYANOBACTERIA**		
** Chroococcales**		
** Chroococcaceae**		
** *Chlorogloea* Wille**		[42]
*Chlorogloea endophytica* Howe		
** *Chroococcus* Nägeli**		
*Chroococcus* minor (Kützing) Nägeli		[42]
** *Entophysalis* Kützing**		
*Entophysalis conferta* (Kützing) Drouet & Daily		[42]
*Entophysalis granulosa* Kützing		[42]
** *Johannesbaptistia* G.De Toni**		
*Johannesbaptistia primaria* (N.L.Gardner) G.De Toni		[42]
** *Limnococcus* (Komárek & Anagnostidis) Komárková, Jezberová, O.Komárek & Zapomelová**		
*Limnococcus limneticus* (Lemmermann) Komárková, Jezberová, O.Komárek & Zapomelová	*Chroococcus limneticus* Lemmermann	[42,62]
** Gomphosphaeriaceae**		
** *Gomphosphaeria* Kützing**		
*Gomphosphaeria aponina* Kützing		[42,63]
** Microcystaceae**		
** *Aphanocapsa* Nägeli**		
*Aphanocapsa litoralis* Hansgirg		[42]
*Aphanocapsa marina* Hansgirg		[42]
*Aphanocapsa reinboldii* (Richter) Komárek & Anagnostidis	*Microcystis reinboldii* (Richter) Forti	[42]
** *Merismopedia* Meyen**		
*Merismopedia glauca* (Ehrenberg) Kützing		[42]
** Pleurocapsaceae**		
** *Chamaecalyx* Komárek & Anagnostidis**		
*Chamaecalyx swirenkoi* (Schirschoff) Komárek & Anagnostidis		[42]
** *Dermocarpella* Lemmermann**		
*Dermocarpella hemisphaerica* (Lemmermann) Lemmermann		[42]
*Dermocarpella prasina* (Reinsch) Komárek & Anagnostidis		[42]
** *Hyella* É.Bornet & C.Flahault**		
*Hyella caespitosa* Bornet & Flahault		[42]
** *Stanieria* J.Komárek & K.Anagnostidis**		
*Stanieria sphaerica* (Setchell & N.L.Gardner) Anagnostidis & Pantazidou		[42]
** *Tryponema* A.Ercegovic**		
*Tryponema endolithicum* Ercegovic		[42]
** Chroococcidiopsidales**		
** Aliterellaceae**		
** *Gloeocapsopsis* Geitler ex Komárek**		
*Gloeocapsopsis crepidinum* (Thuret) Geitler ex Kom á rek		[42]
** Leptolyngbyales**		
** Leptolyngbyaceae**		
** *Heteroleibleinia* (Geitler) Hoffmann**		
*Heteroleibleinia infixa* (Frémy) Anagnostidis et Komárek	*Lyngbya infixa* Frémy	[42]
** *Leptolyngbya* Anagnostidis & Komárek**		
*Leptolyngbya gracilis* (Lindstedt) Anagnostidis & Komárek	*Phormidium gracile* (Rabenhorst ex Gomont) Anagnostidis	[42]
*Leptolyngbya rivulariarum* (Gomont) Anagnostidis & Komárek	*Lyngbya rivulariarum* Gomont	[42,63]
** *Phormidesmis* Turicchia, Ventura, Komárková & Komárek**		
*Phormidesmis mollis* (Gomont) Turicchia, Ventura, Komárková & Komárek	*Phormidium molle* Gomont	[42,63]
** *Planktolyngbya* Anagnostidis & Komárek**		
*Planktolyngbya limnetica* (Lemmermann) Komárková-Legnerová & Cronberg	*Lyngbya limnetica* Lemmermann	[42,63]
** Trichocoleusaceae**		
** *Trichocoleu*s Anagnostidis**		
*Trichocoleus tenerrimus* (Gomont) Anagnostidis	*Microcoleus tenerrimus* Gomont	[42]
** Nostocales**		
** Aphanizomenonaceae**		
** *Gloeotrichia* J.Agardh ex Bornet & Flahault**		
*Gloeotrichia intermedia* (Lemmermann) Geitler		[42,63]
** *Hydrocoryne* H.Schwabe ex É.Bornet & C.Flahault**		
*Hydrocoryne enteromorphoides* (Bornet & Flahault) Umezaki & M.Watanabe	*Hormothamnion enteromorphoides* Grunow ex Bornet & Flahault	[42,63]
*Hydrocoryne soluta* (Bornet & Grunow) I.Umezaki	*Hormothamnion solutum* Bornet & Grunow	[42,63]
** Hapalosiphonaceae**		
** *Mastigocoleus* Lagerheim ex É.Bornet & C.Flahault**		
*Mastigocoleus testarum* Lagerheim ex Bornet & Flahault		[42,63]
** Nostocaceae**		
** *Nostoc* Vaucher ex Bornet & Flahault**		
*Nostoc commune* Vaucher ex Bornet & Flahault		[42,63]
** *Richelia* J.Schmidt**		
*Richelia intracellularis* J.Schmidt		[42,63]
** Rivulariaceae**		
** *Calothrix* C.Agardh ex Bornet & Flahault**		
*Calothrix aeruginea* Thuret ex Bornet & Flahault		[42,63]
*Calothrix aeruginosa* Woronichin		[42]
*Calothrix confervicola* C.Agardh ex Bornet & Flahault		[42]
*Calothrix contarenii* Bornet & Flahault		[42,63]
*Calothrix nidulans* Setchell & N.L.Gardner		[42,63]
*Calothrix parietina* Thuret ex Bornet & Flahault		[42]
*Calothrix pulvinata* C.Agardh ex Bornet & Flahault		[42,63]
*Calothrix scopulorum* C.Agardh ex Bornet & Flahault		[42,63]
** *Kyrtuthrix* Ercegovic**		
*Kyrtuthrix maculans* (Gomont) I.Umezaki		[42,63]
** *Microchaete* Thuret ex Bornet & C.Flahault**		
*Microchaete tapahiensis* Setchell		[42,63]
*Microchaete vitiensis* Askenasy ex Bornet & Flahault		[42,63]
** *Rivularia* C.Agardh ex Bornet & Flahault**		
*Rivularia atra* f. *hemisphaerica* (Bornet & Flahault) Kossinskaja		[42,63]
*Rivularia atra* var. *confluens* Bornet		[42,63]
*Rivularia australis* Harvey ex Bornet & Flahault		[42,63]
** Scytonemataceae**		
** *Brachytrichia* Zanardini ex Bornet & Flahault**		
*Brachytrichia lloydii* (P.Crouan & H.Crouan) P.C.Silva	*Brachytrichia balani* Bornet & Flahault	[42,63]
*Brachytrichia quoyi* Bornet & Flahault		[42,63]
** *Scytonema* C.Agardh ex É.Bornet & C.Flahault**		
*Scytonema ocellatum* Lyngbye ex Bornet & Flahault		[42]
** *Scytonematopsis* Kisseleva**		
*Scytonematopsis crustacea* (Thuret ex Bornet & Flahault) Kováčik & Komárek	*Calothrix crustacea* Thuret ex Bornet & Flahault	[42]
*Scytonematopsis pilosa* (Bornet & Flahault) Umezaki & M.Watanabe	*Calothrix pilosa* Harvey ex Bornet & Flahault	[42,63]
** Oscillatoriales**		
** Coleofasciculaceae**		
** *Coleofasciculus* M.A.Siegesmund, J.R.Johansen & T.Friedl**		
*Coleofasciculus chthonoplastes* (Gomont) M.Siegesmund, J.R.Johansen & T.Friedl	*Microcoleus chthonoplastes* Thuret ex Gomont	[42]
** Microcoleaceae**		
** *Leibleinia* (M.Gomont) L.Hoffman**		
*Leibleinia epiphytica* (Hieronymus) Compère	*Lyngbya epiphytica* Hieronymus	[42,63]
** *Lyngbya* C.Agardh ex Gomont**		
*Lyngbya aestuarii* Liebman ex Gomont		[42,63]
*Lyngbya agardhii* Gomont		[42,63]
*Lyngbya confervoides* C.Agardh ex Gomont		[42,63]
*Lyngbya lutea* Gomont	*Porphyrosiphon luteus* (Gomont) Anagnostidis & Komárek	[42,63]
*Lyngbya majuscula* Harvey ex Gomont		[42,63]
*Lyngbya martensiana* f. *tenuivaginata* Gomont ex Forti		[42,63]
*Lyngbya martensiana* Meneghini ex Gomont		[42,63]
*Lyngbya meneghiniana* Gomont		[42,63]
*Lyngbya semiplena* J.Agardh ex Gomont		[42,63]
*Lyngbya sordida* Gomont		[42,63]
** *Planktothrix* Anagnostidis & Komárek**		
*Planktothrix isothrix* (Skuja) Komárek & Komárková	*Oscillatoria agardhii* Gomont	[42,63]
** *Symploca* Kützing ex Gomont**		
*Symploca hydnoides* Kützing ex Gomont		[42,63]
** Oscillatoriaceae**		
** *Blennothrix* Kützing ex Anagnostidis & Komárek**		
*Blennothrix cantharidosma* (Gomont) Anagnostidis & Komárek	*Hydrocoleum cantharidosmum* (Montagne) Gormont)	[42,63]
*Blennothrix lyngbyacea* (Kützing ex Gomont) Anagnostidis & Komárek	*Hydrocoleum lyngbyaceum* Kützing ex Gomont	[42,63]
** *Oscillatoria* Vaucher ex Gomont**		
*Oscillatoria bonnemaisonii* P.Crouan & H.Crouan ex Gomont		[42,63]
*Oscillatoria corallinae* Gomont	*Phormidium corallinae* (Gomont) Anagnostidis & Komárek	[42,63]
*Oscillatoria indica* P.C.Silva		[42,48]
*Oscillatoria limosa* C.Agardh ex Gomont		[42,64]
*Oscillatoria margaritifera* Kützing ex Gomont		[42,63]
*Oscillatoria miniata* Hauck ex Gomont		[42,63,64]
*Oscillatoria princeps* Vaucher ex Gomont		[42,63]
*Oscillatoria simplicissima* Gomont	*Phormidium simplicissimum* (Gomont) Anagnostidis & Komárek	[42,63]
*Oscillatoria tenuis* C.Agardh ex Gomont		[42,63]
** *Phormidium* Kützing ex Gomont**		
*Phormidium corium* Gomont		[42,63,64]
*Phormidium feldmannii* Frémy		[42,63]
*Phormidium jadinianum* Gomont		[42]
*Phormidium nigroviride* (Thwaites ex Gomont) Anagnostidis & Komárek		[42,63]
*Phormidium nigrum* (Vaucher ex Gomont) Anagnostidis & Komárek		[42,63]
*Phormidium salinum* (Alten) Anagnostidis & Komárek		[48]
** Pleurocapsales**		
** Dermocarpellaceae**		
** *Dermocarpa* P.Crouan & H.Crouan**		
*Dermocarpa acervata* (Setchell & Gardner) Pham-Hoàng Hô		[42]
** Hydrococcaceae**		
** *Hydrococcus* Kützing**		
*Hydrococcus rivularis* Kützing		[42]
** Spirulinales**		
** Spirulinaceae**		
** *Spirulina* Turpin ex Gomont**		
*Spirulina major* Kützing ex Gomont		[42,63,64]
*Spirulina subsalsa* Oersted ex Gomont		[42,63]
*Spirulina subtilissima* Kützing ex Gomont		[42,63]
*Spirulina tenerrima* Kützing ex Gomont		[42,63]
** Pseudanabaenales**		
** Pseudanabaenaceae**		
** *Pseudanabaena* Lauterborn**		
*Pseudanabaena limnetica* (Lemmermann) Komárek		[42]
**RHODOPHYTA**		
** Acrochaetiales**		
** Acrochaetiaceae**		
** *Acrochaetium* Nägeli**		
*Acrochaetium barbadense* (Vickers) Børgesen	*Acrochaetium occidentale* Børgesen	[14,27,38,41,42]
*Acrochaetium catenulatum* M.Howe		[14,27,38,41,42,64]
*Acrochaetium chaetomorphae* (Tanaka & Pham-Hoàng Hô) Heerebout	*Erythrocladia chaetomorphae* T.Tanaka & Pham-Hoàng Hô	[14,41,65]
*Acrochaetium colaconemoides* Pham-Hoàng Hô		[14,41,42]
*Acrochaetium crassipes* (Børgesen) Børgesen		[14,41]
*Acrochaetium erectum* Børgesen		[41,42]
*Acrochaetium liagorae* Børgesen		[27,38,41,42]
*Acrochaetium microscopicum* (Nägeli ex Kützing) Nägeli		[42]
*Acrochaetium phuquocense* Pham-Hoàng Hô		[14,41,42,66]
*Acrochaetium polysporum* M.Howe		[42]
*Acrochaetium pseudoerectum* Pham-Hoàng Hô		[14,38,41,42,66]
*Acrochaetium pulchellum* Børgesen		[14,41,42]
*Acrochaetium sanctaemariae* (Darbishire) Hamel		[14,38,41,42]
*Acrochaetium sanctithomae* Børgesen		[38,41,42]
*Acrochaetium secundatum* (Lyngbye) Nägeli	*Acrochaetium virgatulum* (Harvey) Batters	[14,41,42]
*Acrochaetium subseriatum* Børgesen		[14,38,41,42]
** *Liagorophila* Yamada**		
*Liagorophila endophytica* Yamada	*Acrochaetium yamadae* (Garbary) Y.Lee & I.K.Lee	[38,42]
** Ahnfeltiales**		
** Ahnfeltiaceae**		
** *Ahnfeltia* E.M.Fries**		
*Ahnfeltia plicata* (Hudson) E.M.Fries		[41,42]
** Bangiales**		
** Bangiaceae**		
** *Bangia* Lyngbye**		
*Bangia fuscopurpurea* (Dillwyn) Lyngbye		[14,38,41,42]
*Bangia tanakae* Pham-Hoàng Hô	*Bangia tanakai* Pham H.H.	[14,38,41,42]
** *Phycocalidia* Santiañez & M.J.Wynne**		
*Phycocalidia tanegashimensis* (I.Shinmura) Santiañez		[54]
*Phycocalidia suborbiculata* (Kjellman) Santiañez & M.J.Wynne	*Porphyra suborbiculata* Kjellman, *Pyropia suborbiculata* (Kjellman) J.E.Sutherland, H.G.Choi, M.S.Hwang & W.A.Nelson	[14,38,41,42,67]
** *Porphyra* C.Agardh**		
*Porphyra tanakae* Pham Hoang-Ho	*Porphyra tanaka* Pham H.H.	[41,42]
** *Pyropia* J.Agardh**		
*Pyropia pseudolobata* (L.-E.Yang, J.Brodie & Q.-Q.Lu) Santiañez & M.J.Wynne	*Porphyra suborbiculata* Kjellman	[42,67,68]
*Pyropia vietnamensis* (Tak.Tanaka & P.H.Hô) J.E.Sutherland & Monotilla	*Porphyra vietnamensis* Tak.Tanaka & P.H.Hô	[38,41,69]
** Bonnemaisoniales**		
** Bonnemaisoniaceae**		
** Asparagopsis Montagne**		
*Asparagopsis taxiformis* (Delile) Trevisan		[14,27,38,41,64,70,71,72]
** Ceramiales**		
** Callithamniaceae**		
** *Aglaothamnion* Feldmann-Mazoyer**		
*Aglaothamnion cordatum* (Børgesen) Feldmann-Mazoyer	*Aglaothamnion neglectum* Feldmann-Mazoyer	[27,38,41,42]
** *Callithamnion* Lyngbye**		[14,38,41,42]
*Callithamnion ramosissimum* N.L.Gardner		[42]
** *Crouania* J.Agardh**		
*Crouania attenuata* (C.Agardh) J.Agardh		[27,38,41,42]
** *Gymnothamnion* J.Agardh**		
*Gymnothamnion elegans* (Schousboe ex C.Agardh) J.Agardh		[14,38,41,42]
** *Spyridia* Harvey**		
*Spyridia filamentosa* (Wulfen) Harvey		[38,41,42,64,67]
*Spyridia hypnoides* (Bory) Papenfuss		[38,41,42,64,67]
** Ceramiaceae**		
** *Antithamnion* Nägeli**		
*Antithamnion antillanum* Børgesen		[38,41,64]
*Antithamnion erucacladellum* R.E.Norris		[38,41,42,64]
** *Antithamnionella* Lyle**		
*Antithamnionella basispora* (Tokida & Inaba) Cormaci & G.Furnari	*Antithamnion basisporum* Tokida & Inaba	[14,38,41,42]
*Antithamnionella breviramosa* (E.Y.Dawson) Wollaston		[41]
*Antithamnionella graeffei* (Grunow) Athanasiadis		[27,41,42]
*Antithamnionella spirographidis* (Schiffner) E.M.Wollaston	*Antithamnion spirographidis* Schiffner	[41,42,63]
** *Centroceras* Kützing**		
*Centroceras clavulatum* (C.Agardh) Montagne		[41,42,68,72]
*Centroceras gasparrinii* (Meneghini) Kützing	*Centroceras inerme* Kützing	[14,38,41,42,64]
** *Ceramium* Roth**		
*Ceramium aduncum* Nakamura		[38,41,42,64]
*Ceramium borneense* Weber Bosse		[41,42,63]
*Ceramium cimbricum* H.E.Petersen	*Ceramium fastigiatum* Harvey	[14,38,41,42,64]
*Ceramium cingulatum* Weber Bosse		[14,38,41,42,64]
*Ceramium clarionense* Setchell & N.L.Gardner		[14,38,41,42]
*Ceramium codii* (H.Richards) Mazoyer		[41,42,63]
*Ceramium deslongchampsii* Chauvin ex Duby		[41,42,63]
*Ceramium diaphanum* (Lightfoot) Roth	*Ceramium gracillimum* (Kützing) Zanardini	[41,42,63]
*Ceramium macilentum* J.Agardh	*Ceramium mazatlanense* E.Y.Dawson	[27,41,42,64,68,72]
*Ceramium maryae* Weber Bosse		[14,38,41,42]
*Ceramium procumbens* Setchell & N.L.Gardner		[38,41,42,64]
*Ceramium tenerrimum* (G.Martens) Okamura		[41,42,63]
*Ceramium vagans* P.C.Silva		[41,42,63]
*Ceramium vietnamense* Pham-Hoàng Hô		[14,38,41,42]
*Ceramium zacae* Setchell & N.L.Gardner		[41,42,63]
** *Corallophila* Weber-van Bosse**		
*Corallophila bella* (Setchell & Gardner) R.E.Norris		[27,38,41,42]
*Corallophila howei* (Weber Bosse) R.E.Norris		[14,38,41,42]
*Corallophila huysmansi*i (Weber Bosse) R.E.Norris	*Ceramium huysmansii* Weber Bosse	[14,27,38,41,42]
*Corallophila kleiwegii* Weber Bosse	*Corallophila apiculata* (Yamada) R.E.Norris	[27,41,42,64]
** *Gayliella* T.O.Cho, L.M.McIvor & S.M.Boo**		
*Gayliella fimbriata* (Setchell & N.L.Gardner) T.O.Cho & S.M.Boo	*Ceramium fimbriatum* Setchell & N.L.Gardner	[14,38,41,42]
*Gayliella flaccida* (Harvey ex Kützing) T.O.Cho & L.M.McIvor	*Ceramium flaccidum* (Harvey ex Kützing) Ardissone	[27]
*Gayliella mazoyerae* T.O.Cho, Fredericq & Hommersand	*Ceramium gracillimum* var. *byssoideum* Mazoyer	[38,41,42]
*Gayliella taylorii* (E.Y.Dawson) T.O.Cho & S.M.Boo	*Ceramium taylorii* E.Y.Dawson	[14,38,41,42]
** *Reinboldiella* De Toni**		
*Reinboldiella warburgii* (Heydrich) Yoshida & Mikami		[41,42,63]
** Delesseriaceae**		
** *Acrosorium* Zanardini ex Kützing**		
*Acrosorium polyneurum* Okamura		[41,42,63]
** *Branchioglossum* Kylin**		
*Branchioglossum prostratum* C.W.Schneider		[27,38,41,42]
** *Caloglossa* (Harvey) G.Martens**		
*Caloglossa beccarii* (Zanardini) De Toni		[42,73]
*Caloglossa bengalensis* (G.Martens) R.J.King & Puttock	*Caloglossa adnata* (Zanardini) De Toni	[41,42,63]
*Caloglossa continua* (Okamura) R.J.King & Puttock		[41,42,63]
*Caloglossa leprieurii* (Montagne) G.Martens		[14,38,41,42]
*Caloglossa ogasawaraensis* Okamura		[41,42,63]
*Caloglossa saigonensis* Tanaka & Pham-Hoàng Hô		[41,42,74]
*Caloglossa stipitata* E.Post		[41,42,63]
** *Claudea* J.V.Lamouroux**		
*Claudea batanensis* Tanaka		[38,41,42]
** *Cottoniella* Børgesen**		
*Cottoniella filamentosa* (M.Howe) Børgesen		[41,42,63]
** *Dasya* C.Agardh**		
*Dasya anastomosans* (Weber Bosse) M.J.Wynne	*Dasyopsis pilosa* Weber Bosse; *Dasya pilosa* (Weber Bosse) A.J.K.Millar	[14,38,41,42]
*Dasya crouaniana* J.Agardh		
*Dasya pedicellata* (C.Agardh) C.Agardh	*Dasya baillouviana* (S.G.Gmelin) Montagne	[14,38,41,42]
*Dasya scoparia* Harvey		[41,42,63]
** *Dictyurus* Bory de Saint-Vincent**		
*Dictyurus occidentalis* J.Agardh		[41,42,63]
** *Hypoglossum* Kützing**		
*Hypoglossum attenuatum* N.L.Gardner		[14,38,41,42,64]
*Hypoglossum barbatum* Okamura		[41,42,63]
*Hypoglossum simulans* M.J.Wynne, I.R.Price & D.L.Ballantine		[41,42,63]
** *Martensia* K.Hering**		
*Martensia flabelliformis* Harvey ex J.Agardh	*Neomartensia flabelliformis* (Harvey ex J.Agardh) Yoshida & Mikami	[41,42,63]
*Martensia fragilis* Harvey		[38,41,42]
** *Nitophyllum* Greville**		
*Nitophyllum adhaerens* M.J.Wynne		[27,41,42]
** *Taenioma* J.Agardh**		
*Taenioma perpusillum* (J.Agardh) J.Agardh		[41,42,63]
** *Zellera* G.Martens**		
*Zellera tawallina* G.Martens		[55]
** Rhodomelaceae**		
** *Acanthophora* J.V.Lamouroux**		
*Acanthophora muscoides* (Linnaeus) Bory		[38,41,42,64,67,68]
*Acanthophora spicifera* (M.Vahl) Børgesen		[27,41,42,64,68,72]
** *Acrocystis* Zanardini**		
*Acrocystis nana* Zanardini		[14,38,41,42]
** *Amansia* J.V.Lamouroux**		
*Amansia glomerata* C.Agardh	*Melanamansia glomerata* (C. Agardh) R.E. Norris	[38,41,42,75]
*Amansia rhodantha* (Harvey) J.Agardh		[41,42,63]
** *Bostrychia* Montagne**		
*Bostrychia moritziana* (Sonder ex Kützing) J.Agardh		[63]
*Bostrychia radicans* (Montagne) Montagne		[14,38,41,42]
*Bostrychia tenella* (J.V.Lamouroux) J.Agardh		[38,41,42,67]
** *Bryocladia* F.Schmitz**		
*Bryocladia cervicornis* (Kützing) F.Schmitz		[14,38,41,42]
** *Chondria* C.Agardh**		
*Chondria armata* (Kützing) Okamura		[41,42,63]
*Chondria baileyana* (Montagne) Harvey		[14,38,41,42]
*Chondria dangeardii* E.Y.Dawson		[14,27,38,41,42]
*Chondria repens* Børgesen		[14,38,41,42,64]
*Chondria ryukyuensis* Yamada		[41,42,63]
*Chondria simpliciuscula* Weber Bosse		[27,38,41,42]
** *Chondrophycus* (J.Tokida & Y.Saito) Garbary & J.T.Harper**		
*Chondrophycus articulatus* (C.K.Tseng) K.W.Nam	*Laurencia articulata* C.K.Tseng	[14,38,41,42]
*Chondrophycus cartilagineus* (Yamada) Garbary & J.T.Harper	*Laurencia cartilaginea* Yamada	[14,38,41,42]
*Chondrophycus tronoi* (E.Ganzon-Fortes) K.W.Nam		[47]
*Chondrophycus undulatus* (Yamada) Garbary & Harper		[41,42,63]
*Chondrophycus verticillatus* (J.F.Zhang & B.M.Xia) K.W.Nam		[41,42,63]
** *Endosiphonia* Zanardini**		
*Endosiphonia horrida* (C.Agardh) P.C.Silva		[41,42,63]
** *Epizonaria* Díaz-Tapia & Maggs**		
*Epizonaria prostrata* (Harvey) Díaz-Tapia & Maggs	*Lophosiphonia prostrata* (Harvey) Falkenberg	[27,38,41,42]
** *Eutrichosiphonia* Savoie & G.W.Saunders**		
*Eutrichosiphonia tapinocarpa* (Suringar) D.E.Bustamante & T.O.Cho	*Polysiphonia tapinocarpa* Suringar	[41,42,63]
** *Exophyllum* Weber-van Bosse**		
*Exophyllum wentii* Weber Bosse		[41,42,63]
** *Herposiphonia* Nägeli**		
*Herposiphonia caespitosa* C.K.Tseng		[41,42,63]
*Herposiphonia crassa* Hollenberg		[27,38,41,42]
*Herposiphonia delicatula* Hollenberg		[27,38,41,42]
*Herposiphonia insidiosa* (Greville ex J.Agardh) Falkenberg		[14,38,41,42]
*Herposiphonia parca* Setchell		[27,41,42,64]
*Herposiphonia tenella* (C.Agardh) Ambronn	*Herposiphonia secunda* f. *tenella* (C.Agardh) M.J.Wynne	[14,27,38,41,42]
*Herposiphonia vietnamica* Pham-Hoàng Hô		
** *Laurencia* J.V.Lamouroux**		
*Laurencia brachyclados* Pilger		[41,42,63]
*Laurencia caduciramulosa* Masuda & S.Kawaguchi		[41,42,63]
*Laurencia calliclada* Masuda		[41,42,63]
*Laurencia corymbosa* J.Agardh		[14,38,41,42]
*Laurencia decumbens* Kützing		[38,41,42]
*Laurencia dendroidea* J.Agardh	*Laurencia majuscula* (Harvey) A.H.S.Lucas	[38,41,42,64]
*Laurencia filiformis* (C.Agardh) Montagne		[41,42,63]
*Laurencia galtsoffii* M.Howe		[41,42,63]
*Laurencia heteroclada* Harvey		[41,42,63]
*Laurencia intricata* J.V.Lamouroux		[41,42,63]
*Laurencia lageniformis* Masuda & Suzuki		[41,42,63]
*Laurencia mariannensis* Yamada		[41,42,63]
*Laurencia microcladia* Kützing		[14,38,41,42]
*Laurencia nangii* Masuda		[28,38,41,42]
*Laurencia natalensis* Kylin		[41,42,63]
*Laurencia nidifica* J.Agardh		[41,42,63]
*Laurencia obtusa* (Hudson) J.V.Lamouroux		[38,41,42]
*Laurencia obtusa* var. *densa* Yamada		[41,42,63]
*Laurencia pinnata* Yamada		[38,41,42]
*Laurencia silvae* J.F.Zhang & B.M.Xia	*Laurencia fasciculata* J.F.Zhang & B.M.Xia	[41,42,63]
*Laurencia similis* K.W.Nam & Y.Saito		[41,42,63]
*Laurencia tenera* C.K.Tseng		[14,38,41,42]
*Laurencia tropica* Yamada		[38,41,42,67]
** *Leveillea* Decaisne**		
*Leveillea jungermannioides* (Hering & G.Martens) Harvey		[14,27,41,42,67]
** *Lophosiphonia* Falkenberg**		
*Lophosiphonia obscura* (C.Agardh) Falkenberg		[38,41,42]
** *Melanothamnus* Bornet & Falkenberg**		
*Melanothamnus ferulaceus* (Suhr ex J.Agardh) Díaz-Tapia & Maggs	*Neosiphonia ferulacea* (Suhr ex J.Agardh) S.M.Guimarães & M.T.Fujii	[41,42,63]
*Melanothamnus harlandii* (Harvey) Díaz-Tapia & Maggs	*Neosiphonia harlandii* (Harvey) M.S.Kim & I.K.Lee	[38,41,42]
*Melanothamnus sparsus* (Setchell) Díaz-Tapia & Maggs	*Neosiphonia sparsa* (Setchell) I.A.Abbott	[27,38,41,42]
*Melanothamnus sphaerocarpus* (Børgesen) Díaz-Tapia & Maggs	*Neosiphonia sphaerocarpa* (Børgesen) M.-S.Kim & I.K.Lee	[27,41,42,64]
*Melanothamnus tongatensis* (Harvey ex Kützing) Díaz-Tapia & Maggs	*Neosiphonia tongatensis* (Harvey ex Kützing) M.-S.Kim & I.K.Lee	[14,41,42,64]
*Melanothamnus upolensis* (Grunow) Díaz-Tapia & Maggs	*Neosiphonia upolensis* (Grunow) M.S.Kim & Boo	[41,42,63]
*Melanothamnus infestans* (Harvey) Huisman	*Polysiphonia infestans* Harvey	[41]
*Melanothamnus thailandicus* (N.Muangmai & C.Kaewsuralikhit) Díaz-Tapia & Maggs		[51]
** *Neurymenia* J.Agardh**		
*Neurymenia fraxinifolia* (Mertens ex Turner) J.Agardh		[41,42,63]
** *Odonthalia* Lyngbye**		
*Odonthalia corymbifera* (S.G.Gmelin) Greville		[41,42,63]
** *Ohelopapa* F.Rousseau, Martin-Lescanne, Payri & L.Le Gall**		
*Ohelopapa flexilis* (Setchell) F.Rousseau, Martin-Lescanne, Payri & L.Le Gall		[39,42]
** *Palisada* K.W.Nam**		
*Palisada concreta* (A.B.Cribb) K.W.Nam		[38,41,42]
*Palisada intermedia* (Yamada) K.W.Nam		[41,42,63]
*Palisada parvipapillata* (C.K.Tseng) K.W.Nam		[38,41,42]
*Palisada perforata* (Bory) K.W.Nam		[41,42,63]
*Palisada thuyoides* (Kützing) Cassano, Sentíes, Gil-Rodríguez & M.T.Fujii		[38,41,42]
*Palisada yamadana* (M.Howe) K.W.Nam		[38,41,42]
** *Polysiphonia* Greville**		
*Polysiphonia coacta* C.K.Tseng		[14,38,41,42]
*Polysiphonia fragilis* Suringar		[14,38,41,42]
*Polysiphonia kampsaxii* Børgesen		[41,42,63]
*Polysiphonia nhatrangense* Pham-Hoàng Hô		[14,38,41,42]
*Polysiphonia poko* Hollenberg	*Neosiphonia poko* (Hollenberg) I.A.Abbott	[38,41,42]
*Polysiphonia scopulorum* Harvey		[27,38,41,42,64,69]
*Polysiphonia sertularioides* (Grateloup) J.Agardh		[41,42,63]
*Polysiphonia subtilissima* Montagne	*Neosiphonia subtilissima* (Montagne) M.S. Kim & I.K. Lee	[14,27,38,41,42]
*Polysiphonia villum* J.Agardh	*Polysiphonia scopulorum* var. *villum* (J.Agardh) Hollenberg	[38,41,42,64]
** *Rodriguezella* F.Schmitz**		
*Rodriguezella hongngai* Pham-Hoàng Hô		[14,38,41,42]
** *Symphyocladia* Falkenberg**		
*Symphyocladia marchantioides* (Harvey) Falkenberg		[41,42,63]
** *Tayloriella* Kylin**		
*Tayloriella dictyurus* (J.Agardh) Kylin		[41,42,63]
** *Tolypiocladia* F.Schmitz**		
*Tolypiocladia calodictyon* (Harvey ex Kützing) P.C.Silva		[41,42,63]
*Tolypiocladia glomerulata* (C.Agardh) F.Schmitz		[14,27,41,42,67]
** *Vertebrata* S.F.Gray**		
*Vertebrata reptabunda* (Suhr) Díaz-Tapia & Maggs	*Lophosiphonia reptabunda* (Suhr) Kylin	[14,38,41,42]
** *Womersleyella* Hollenberg**		
*Womersleyella herpa* (Hollenberg) R.E.Norris	*Polysiphonia herpa* Hollenberg	[14,38,41,42]
** Wrangeliaceae**		
** Anotrichium Nägeli**		
*Anotrichium barbatum* Nägeli		[41,42,63]
*Anotrichium tenue* (C.Agardh) Nägeli	*Anotrichium tenue* var. *thyrsigerum* (Thwaites ex Harvey) H.S.Kim & I.K.Lee	[27,41,42,64]
** *Diplothamnion* A.B.Joly & Yamaguishi**		
*Diplothamnion jolyi* C.Hoek		[41,42,64]
** *Griffithsia* C.Agardh**		
*Griffithsia heteromorpha* Kützing		[38,41,42,64]
*Griffithsia japonica* Okamura		[14,27,38,41,42,64,67]
*Griffithsia metcalfii* C.K. Tseng		[38,41,42,64]
** *Haloplegma* Montagne**		
*Haloplegma duperreyi* Montagne		[41,42,63]
** *Monosporus* Solier**		
*Monosporus pedicellatus* var. *tenuis* (Feldmann-Mazoyer) Huisman & Kraft	*Neomonospora pedicellata* var. *tenuis* Feldmann-Mazoyer	[38,41,42]
** *Pleonosporium* Nägeli**		
*Pleonosporium borreri* (Smith) Nägeli		[14,38,41,42]
** *Spongoclonium* Sonder**		
*Spongoclonium caribaeum* (Børgesen) M.J.Wynne	*Mesothamnion caribaeum* Børgesen	[41,42,63]
** *Wrangelia* C.Agardh**		
*Wrangelia argus* (Montagne) Montagne		[14,38,41,42]
*Wrangelia dumontii* (E.Y.Dawson) I.A.Abbott		[27,38,41,42]
*Wrangelia tanegana* Harvey		[38,41,42]
** Colaconematales**		
** Colaconemataceae**		
** *Colaconema* Batters**		
*Colaconema gracile* (Børgesen) Ateweberhan & Prud’homme	*Acrochaetium gracile* Børgesen	[14,27,38,41,42]
*Colaconema gracile* var. *vietnamense* Pham Hoang Ho		[41,42,63]
*Colaconema hallandicum* (Kylin) Afonso-Carrillo, Sanson, Sangil & Diaz-Villa		[38,41,42]
*Colaconema hypneae* (Børgesen) A.A.Santos & C.W.N.Moura		[38,41,42,64]
*Colaconema robustum* (Børgesen) Huisman & Woelkerling	*Acrochaetium robustum* Børgesen; *Audouinella robusta* (Børgesen) Garbary	[14,38,41,42]
*Colaconema thuretii* (Bornet) P.W.Gabrielson		[38,41,42]
** Compsopogonales**		
** Compsopogonaceae**		
** *Compsopogon* Montagne**		
*Compsopogon caeruleus* (Balbis ex C.Agardh) Montagne		[41,42,63]
** Corallinales**		
** Corallinaceae**		
** *Corallina* Linnaeus**		
*Corallina officinalis* Linnaeus		[41,42,63]
*Corallina pilulifera* Postels & Ruprecht		[41,42,63]
** *Jania* J.V.Lamouroux**		
*Jania acutiloba* (Decaisne) J.H.Kim, Guiry & H.-G.Choi		[38,41,42]
*Jania cultrata* (Harvey) J.H.Kim, Guiry & H.-G.Choi	*Cheilosporum cultratum* (Harvey) Areschoug	
*Jania longiarthra* E.Y.Dawson		[14,38,41,42]
*Jania micrarthrodia* J.V.Lamouroux		[38,41,42,64]
*Jania pedunculata* var. *adhaerens* (J.V.Lamouroux) A.S.Harvey, Woelkerling & Rviers	*Jania adhaerens* J.V.Lamouroux; *Jania decussatodichotoma* (Yendo) Yendo; *Jania capillacea* Harvey	[27,41,42,64,68,72]
*Jania pumila* J.V.Lamouroux		[14,27,38,41,42]
*Jania rubens* (Linnaeus) J.V.Lamouroux		[14,38,41,42]
*Jania spectabilis* (Harvey ex Grunow) J.H.Kim, Guiry & H.-G.Choi		[38,41,42]
*Jania squamata* (Linnaeus) J.H.Kim, Guiry & H.-G.Choi		[41,42,63]
*Jania ungulata* f. *brevior* (Yendo) Yendo		[27,38,41,42]
** *Pneophyllum* Kützing**		
*Pneophyllum confervicola* (Kützing) Y.M.Chamberlain	*Heteroderma minutulum* (Foslie) Foslie); *Melobesia confervicola* (Kützing) Foslie	[38,41,42]
** Hapalidiaceae**		
** *Lithothamnion* Heydrich**		
*Lithothamnion erubescens* f. *subflabellatum* Foslie		[38,41,42]
** Hydrolithaceae**		
** *Hydrolithon* (Foslie) Foslie**		
*Hydrolithon boergesenii* (Foslie) Foslie	*Hydrolithon reinboldii* (Weber Bosse & Foslie) Foslie	[14,38,41,42]
*Hydrolithon farinosum* (J.V.Lamouroux) Penrose & Y.M.Chamberlain		[38,41,42,64]
** Lithophyllaceae**		
** *Amphiroa* J.V.Lamouroux**		
*Amphiroa anceps* (Lamarck) Decaisne		[41,42,63]
*Amphiroa beauvoisii* J.V.Lamouroux		[27,41,42,64]
*Amphiroa echigoensis* Yendo		[41,42,63]
*Amphiroa foliacea* J.V.Lamouroux, Bory & Eudes-Deslongchamps		[14,27,38,41,42,67]
*Amphiroa fragilissima* (Linnaeus) J.V.Lamouroux		[27,41,42,64,68,72]
*Amphiroa valonioides* Yendo		[38,41,42,64]
*Amphiroa crassa* J.V.Lamouroux, Bory & Eudes-Deslongchamps		[41]
** *Lithophyllum* Philippi**		
*Lithophyllum okamurae* Foslie		[14,38,41,42]
*Lithophyllum pygmaeum* (Heydrich) Heydrich		[41,42,63]
** *Titanoderma* Nägeli**		
*Titanoderma pustulatum* (J.V.Lamouroux) Nägeli	*Lithophyllum pustulatum* (J.V.Lamouroux) Foslie	[41,42,63]
** Mastophoraceae**		
** *Mastophora* Decaisne**		
*Mastophora pacifica* (Heydrich) Foslie	*Lithoporella pacifica* (Heyrich) Foslie	[38,41,42]
*Mastophora rosea* (C.Agardh) Setchell		[38,41,42,64,67]
** Mesophyllumaceae**		
** *Melyvonnea* Athanasiadis & D.L.Ballantine**		
*Melyvonnea erubescens* (Foslie) Athanasiadis & D.L.Ballantine		[41,42,63]
** *Mesophyllum* Me.Lemoine**		
*Mesophyllum simulans* (Foslie) Me.Lemoine	*Lithothamnion simulans* (Foslie) Foslie	[41,42,63]
** Porolithaceae**		
** *Harveylithon* A.Rösler, Perfectti, V.Peña & J.C.Braga**		
*Harveylithon samoënse* (Foslie) A.Rösler, Perfectti, V.Peña & J.C.Braga	*Hydrolithon samoënse* (Foslie) Keats & Y.M. Chamberlain	[38,41,42,67]
** *Metagoniolithon* Weber-van Bosse**		
*Metagoniolithon stelliferum* (Lamarck) Ducker		[41,42,63]
** Spongitidaceae**		
** *Neogoniolithon* Setchell & L.R.Mason**		
*Neogoniolithon oblimans* (Heydrich) P.C.Silva		[14,38,41,42]
*Neogoniolithon trichotomum* (Heydrich) Setchell & L.R.Mason		[41,42,63]
** Erythropeltales**		
** Erythrotrichiaceae**		
** *Erythrocladia* Rosenvinge**		
*Erythrocladia irregularis* Rosenvinge		[41,42,63]
** *Erythrotrichia* Areschoug**		
*Erythrotrichia carnea* (Dillwyn) J.Agardh		[14,27,38,41,42]
*Erythrotrichia parietalis* T.Tanaka		[14,38,41,42]
*Erythrotrichia parietalis* var. *majuscula* T.Tanaka & Pham-Hoàng Hô		[41,42,63]
** *Sahlingia* Kornmann**		
*Sahlingia subintegra* (Rosenvinge) Kornmann	*Erythrocladia subintegra* Rosenvinge	[41,42,63]
** Gelidiales**		
** Gelidiaceae**		
** *Gelidium* J.V.Lamouroux**		
*Gelidium corneum* (Hudson) J.V.Lamouroux		[41,42,63]
*Gelidium crinale* (Hare ex Turner) Gaillon		[14,38,41,42]
*Gelidium crinale* var. *perpusillum* Piccone & Grunow		[38,41,42]
*Gelidium divaricatum* G.Martens	*Gelidiophycus divaricatus* (G.Martens) G.H.Boo, J.K.Park & S.M.Boo	[14,38,41,42,76]
*Gelidium fasciculatum* Hamel		[41,42,63]
*Gelidium minusculum* (Weber Bosse) R.E.Norris	*Gelidium pusillum* var. *minusculum* Weber Bosse	[41,42,63]
*Gelidium pulchellum* (Turner) Kützing		[[4,27,38,41,42]
*Gelidium pusillum* (Stackhouse) Le Jolis		[27,41,42,64,68,72]
*Gelidium pusillum* var. *cylindricum* W.R.Taylor		[41]
*Gelidium pusillum* var. *pacificum* W.R.Taylor		[41,42,63]
*Gelidium samoënse* Reinbold		[41,42,63]
*Gelidium spathulatum* (Kützing) Bornet		[14,38,41,42]
*Gelidium vietnamense* Pham-Hoàng Hô		[41,42,63]
** Gelidiellaceae**		
** *Gelidiella* Feldmann & G.Hamel**		
*Gelidiella acerosa* (Forsskål) Feldmann & Hamel		[14,27,38,41,42,67]
*Gelidiella lubrica* (Kützing) Feldmann & Hamel		[14,38,41,42]
** *Millerella* G.H.Boo & S.M.Boo**		
*Millerella myrioclada* (Børgesen) G.H.Boo	*Gelidiella myrioclada* (Børgesen) Feldmann & Hamel	[14,38,41,42]
*Millerella pannosa* (Feldmann) G.H.Boo & L.Le Gall	*Parviphycus pannosus* (Feldmann) G.Furnari	[38,41,42,64]
** *Parviphycus* Santelices**		
*Parviphycus trinitatensis* (W.R.Taylor) M.J.Wynne		[41,53]
*Parviphycus adnatus* (E.Y.Dawson) B.Santelices	*Gelidiella adnata* E.Y.Dawson	[27,38,41,42,53,77]
** *Perronella* G.H.Boo, T.V.Nguyen, J.Y.Kim & S.M. Boo**		
*Perronella gracilis* G.H.Boo, T.V.Nguyen, J.Y.Kim & S.M. Boo		[41,53]
** Pterocladiaceae**		
** *Pterocladia* J.Agardh**		
*Pterocladia heteroplatos* (Børgesen) Umamaheswara Rao & Kaliaperumal	*Gelidium heteroplatos* Børgesen	[41,42,63]
** *Pterocladiella* B.Santelices & Hommersand**		
*Pterocladiella bartlettii* (Taylor) Santelices		[57]
*Pterocladiella maribagoensis* G.H.Boo & P.J.L.Geraldino		[57]
*Pterocladiella musciformis* (W.R.Taylor) G.H.Boo & K.A.Miller		[57]
*Pterocladiella caerulescens* (Kützing) Santelices & Hommersand		[27,41,42,64]
*Pterocladiella caloglossoides* (M.Howe) Santelices		[41,42,63]
*Pterocladiella capillacea* (S.G.Gmelin) Santelices & Hommersand	*Pterocladia capillacea* (S.G. Gmelin) Bornet in Bornet et Thuret *Pterocladia pinnata* (Hudson) Papenfuss	[41,42,63]
*Pterocladiella tenuis* (Okamura) Shimada, Horiguchi & Masuda	*Pterocladia tenuis* Okamura	[41,42,63]
** Gigartinales**		
** Calosiphoniaceae**		
** *Schmitzia* P.C.Silva**		
*Schmitzia japonica* (Okamura) P.C.Silva	*Bertholdia japonica* (Okamura) Segawa	[41,42,63]
** Caulacanthaceae**		
** *Catenella* Greville**		
*Catenella impudica* (Montagne) J.Agardh		[41,42,63]
*Catenella nipae* Zanardini		[41,42,63]
*Catenella subumbellata* C.K.Tseng		[41,42,63]
** *Caulacanthus* Kützing**		
*Caulacanthus ustulatus* (Turner) Kützing		[41,42,63]
** *Montemaria* A.B.Joly & Alveal**		
*Montemaria horridula* (Montagne) A.B.Joly & Alveal		[14,38,41,42]
** Clavicloniaceae**		
** *Antrocentrum* Kraft & Min-Thein**		
*Antrocentrum nigrescens* (Harvey) Kraft & Min-Thein		[41,42,63]
** Cystocloniaceae**		
** *Hypnea* J.V.Lamouroux**		
*Hypnea alopecuroides* Kützing		[41,42,63]
*Hypnea anastomosans* Papenfuss, Lipkin & P.C.Silva		[41,42,63]
*Hypnea cenomyce* J.Agardh		[14,38,41,42]
*Hypnea cervicornis* J.Agardh	*Hypnea boergesenii* T.Tanaka	[14,27,38,41,42]
*Hypnea charoides* J.V.Lamouroux		[41,42,63]
*Hypnea charoides* var. *indica* Weber Bosse		[41,42,63]
*Hypnea cornuta* (Kützing) J.Agardh		[14,38,41,42]
*Hypnea esperi* Bory		[14,27,38,41,42]
*Hypnea flagelliformis* Greville ex J.Agardh		[41,42,63]
*Hypnea hamulosa* (Esper) J.V.Lamouroux		[41,42,63]
*Hypnea japonica* Tanaka		[41,42,63]
*Hypnea nidulans* Setchell		[14,27,38,41,42]
*Hypnea pannosa* J.Agardh		[41,42,68,72]
*Hypnea spinella* (C.Agardh) Kützing		[14,27,38,41,42]
*Hypnea valentiae* (Turner) Montagne		[14,27,38,41,42,67,68]
** Dicranemataceae**		
** *Tylotus* J.Agardh**		
*Tylotus lichenoides* Okamura		[41]
** Dumontiaceae**		
** *Gibsmithia* Doty**		
*Gibsmithia hawaiiensis* Doty		[41,42,63]
** Endocladiaceae**		
** *Gloiopeltis* J.Agardh**		
*Gloiopeltis furcata* (Postels & Ruprecht) J.Agardh		[41,42,63]
*Gloiopeltis tenax* (Turner) Decaisne		[41,42,63]
** Gigartinaceae**		
** *Chondracanthus* Kützing**		
*Chondracanthus acicularis* (Roth) Fredericq	*Gigartina acicularis* (Roth) J.V.Lamouroux	[41,42,63]
*Chondracanthus intermedius* (Suringar) Hommersand		[27,38,41,42,67]
*Chondracanthus tenellus* (Harvey) Hommersand	*Gigartina tenella* Harvey	[41,42,63]
** Phyllophoraceae**		
** *Ahnfeltiopsis* P.C.Silva & DeCew**		
*Ahnfeltiopsis chnoosporoides* (T.Tanaka & Pham-Hoàng Hô) Masuda		[41,42,63]
*Ahnfeltiopsis densa* (J.Agardh) P.C.Silva & DeCew	*Gymnogongrus densus* J.Agardh	[41,42,63]
*Ahnfeltiopsis flabelliformis* (Harvey) Masuda		[27,38,41,42,67,68]
*Ahnfeltiopsis pygmaea* (J.Agardh) P.C.Silva & DeCew		
*Ahnfeltiopsis quinhonensis* (Pham-Hoang Ho) Masuda		[27,28,38,41,42,67]
*Ahnfeltiopsis serenei* (E.Y.Dawson) Masuda		[41,42,63]
** *Besa* Setchell**		
*Besa divaricata* (Holmes) M.S.Calderon & S.M.Boo	*Ahnfeltiopsis divaricata* (Holmes) Masuda	[41,42,63]
** *Gymnogongrus* C.Martius**		
*Gymnogongrus griffithsiae* (Turner) C.Martius		[41,42,63]
*Gymnogongrus johnstonii* (Setchell & N.L.Gardner) E.Y.Dawson		[41,42,63]
** Rhizophyllidaceae**		
** *Portieria* Zanardini**		
*Portieria hornemannii* (Lyngbye) P.C.Silva	*Desmia hornemannii* Lyngbye	[41,42,63]
*Portieria japonica* (Harvey) P.C.Silva		[41,42,63]
** Solieriaceae**		
** *Betaphycus* Doty**		
*Betaphycus gelatinus* (Esper) Doty ex P.C.Silva	*Eucheuma gelatinum* (Esper) J.Agardh	[14,26,38,41,42,78]
** *Eucheuma* J.Agardh**		
*Eucheuma edule* (Kützing) Weber Bosse		[41,42,63]
** *Kappaphycopsis* Dumilag & Zuccarello**		
*Kappaphycopsis cottonii* (Weber Bosse) Dumilag & Zuccarello	*Kappaphycus cottonii* (Weber Bosse) Doty ex H.D.Nguyen & Q.N.Huynh	[41,42,63]
** *Kappaphycus* Doty**		
*Kappaphycus alvarezii* (Doty) L.M.Liao	*Kappaphycus alvarezii* (Doty) Doty ex P.C. Silva	[41,42,63]
*Kappaphycus inermis* (F.Schmitz) Doty ex H.D.Nguyen & Q.N.Huynh		
*Kappaphycus striatus* (F.Schmitz) L.M.Liao	*Kappaphycus striatus* (F. Schmitz) Doty ex P.C. Silva	[38,41,42]
** *Meristotheca* J.Agardh**		
*Meristotheca lysonensis* X.-V.Nguyen, X.-T.Nguyen, Kittle & McDermid		[52]
** *Mimica* Santiañez & M.J.Wynne**		
*Mimica arnoldii* (Weber Bosse) Santiañez & M.J.Wynne	*Eucheuma arnoldii* Weber Bosse	[41,42,63]
** *Solieria* J.Agardh**		
*Solieria robusta* (Greville) Kylin		[41,42,63]
** *Wurdemannia* Harvey**		
*Wurdemannia miniata* (Sprengel) Feldmann & Hamel		[38,41,42,67]
** Gracilariales**		
** Gracilariaceae**		
** *Gracilaria* Greville**		
*Gracilaria arcuata* Zanardini		[14,23,27,38,41,42,79,80]
*Gracilaria articulata* C.F.Chang & B.M.Xia		[23,42,79]
*Gracilaria blodgettii* Harvey		[41,42,63]
*Gracilaria bursa-pastoris* (S.G.Gmelin) P.C.Silva		[41,42,63]
*Gracilaria canaliculata* Sonder		[38,41,42]
*Gracilaria changii* (B.M.Xia & I.A.Abbott) I.A.Abbott, J.Zhang & B.M.Xia	*Hydropuntia changii* (B.M. Xia & I.A. Abbott) M.J. Wynne	[38,42,80]
*Gracilaria chondracantha* (Kützing) A.J.K.Millar	*Gracilaria bangmeiana* J.Zhang & I.A.Abbott	[23,41,42,79]
*Gracilaria confervoides* f. *ecortica* V.M.May		[41,42,63]
*Gracilaria coronopifolia* J.Agardh		[41,42,68,72]
*Gracilaria cuneifolia* (Okamura) I.K.Lee & Kurogi		[38,41,42]
*Gracilaria divergens* (C.Agardh) J.Agardh		[41]
*Gracilaria edulis* (S.G.Gmelin) P.C.Silva	*Hydropuntia edulis* (S.G.Gmelin) Gurgel & Fredericq	[14,27,38,41,42,67,68]
*Gracilaria eucheumatoides* Harvey	*Hydropuntia eucheumatoides* (Harvey) Gurgel & Fredericq	[38,41,42,81]
*Gracilaria firma* C.F.Chang & B.-M.Xia	*Crassiphycus firmus* (C.F.Chang & B.-M.Xia) Gurgel, J.N.Norris & Fredericq	[23,38,41,42,80]
*Gracilaria fisheri* (B.M.Xia & I.A.Abbott) I.A.Abbott, J.Zhang & B.M.Xia	*Hydropuntia fisheri* (B.M.Xia & I.A.Abbott) M.J.Wynne	[38,41,42,80]
*Gracilaria foliifera* (Forsskål) Børgesen		[41,42,63]
*Gracilaria gigas* Harvey		[23,42,79]
*Gracilaria gracilis* (Stackhouse) Steentoft, L.M.Irvine & Farnham		[41,42,63]
*Gracilaria hainanensis* C.F.Chang & B.M.Xia		[23,42,79]
*Gracilaria longirostris* Zhang & Wang		[38,41,42]
*Gracilaria phuquocensis* N.H.Le, N.Muangmai & G.C.Zuccarello	*Gracilaria mammillaris* (Montagne) M.Howe	[49]
*Gracilaria punctata* (Okamura) Yamada		[23,42]
*Gracilaria rubra* C.F.Chang & B.M.Xia		[41]
*Gracilaria salicornia* (C.Agardh) E.Y.Dawson		[27,41,42,64,67,68,72,80]
*Gracilaria spinulosa* (Okamura) Chang & B.-M.Xia		[41,42,68,72]
*Gracilaria stellata* I.A.Abbott, Zhang & B.M.Xia		[41,42,63]
*Gracilaria tenuistipitata* C.F.Chang & B.-M.Xia	*Agarophyton tenuistipitatum* (C.F.Chang et B.-M.Xia) Gurgel, J.N.Norris & Fredericq	[27,38,41,42,67,80]
*Gracilaria tenuistipitata* var. *liui* Zhang & Xia		[41,42,63]
*Gracilaria textorii* (Suringar) Hariot	*Gracilaria textorii* (Suringar) De Toni	[23,41,42,79,80]
*Gracilaria vermiculophylla* (Ohmi) Papenfuss		[41,42,63]
*Gracilaria vieillardii* P.C.Silva		[42,79]
*Gracilaria yamamotoi* Zhang & B.M.Xia		[41,42,63]
** *Gracilariopsis* E.Y.Dawson**		
*Gracilariopsis chorda* (Holmes) Ohmi		[41,42,63]
*Gracilariopsis heteroclada* J.-F.Zhang & B.-M.Xia	*Gracilaria heteroclada* J.F.Zhang & B.M.Xia *Gracilariopsis bailiniae* J.Zhang & B.M.Xia	[41,42,63]
*Gracilariopsis longissima* (S.G.Gmelin) Steentoft, L.M.Irvine & Farnham		[41,42,63]
*Gracilariopsis nganii* Pham-Hoàng Hô		[41,42,63]
*Gracilariopsis nhatrangensis* Nhu Hau Le & S.-M.Lin		[38,41,42]
*Gracilariopsis phanthietensis* Pham-Hoàng Hô		[41,42,63]
*Gracilariopsis rhodotricha* E.Y.Dawson		[14,38,41,42]
** *Hydropuntia* Montagne**		
*Hydropuntia divergens* (B.M.Xia & I.A.Abbott) M.J.Wynne		[41,42,63]
** Halymeniales**		
** Grateloupiaceae**		
** *Dermocorynus* P.Crouan & H.Crouan**		
*Dermocorynus dichotomus* (J.Agardh) Gargiulo, Morabito & Manghisi	*Grateloupia dichotoma* J.Agardh	[41,42,63]
** *Grateloupia* C.Agardh**		
*Grateloupia asiatica* S.Kawaguchi & H.W.Wang		[41,42,63]
*Grateloupia divaricata* Okamura		[14,38,41,42]
*Grateloupia filicina* (J.V.Lamouroux) C.Agardh		[41,42,68,72]
*Grateloupia lithophila* Børgesen		[41,42,63]
*Grateloupia livida* (Harvey) Yamada		[41,42,63]
*Grateloupia phuquocensis* Tanaka & Pham-Hoàng Hô		[41,42,63]
*Grateloupia porracea* Kützing		[41,42,63]
*Grateloupia prolongata* J.Agardh		[41,42,63]
*Grateloupia ramosissima* Okamura		[38,41,42,67]
** *Yonagunia* S.Kawaguchi & M.Masuda**		
*Yonagunia formosana* (Okamura) Kawaguchi & Masuda	*Carpopeltis formosana* Okamura *Prionitis formosana* (Okamura) Kawaguchi & Nguyen H. Dinh	[14,38,41,42,78]
*Yonagunia ligulata* (Harvey ex Kützing) Manghisi, M.Morabito, De Clerck & Le Gall	*Polyopes ligulatus* (Harvey ex Kützing) De Toni	[41,42,63]
*Yonagunia maillardii* (Montagne & Maillardet) Showe M.Lin, Y.-C.Chuang & DeClerck	*Carpopeltis maillardii* (Montagne & Millardet) Chiang	[38,41,42]
** Halymeniaceae**		
** *Cryptonemia* J.Agardh**		
*Cryptonemia undulata* Sonder		[41,42,63]
** *Halymenia* C.Agardh**		
*Halymenia dilatata* Zanardini		[14,27,38,41,42,64,78]
*Halymenia floresii* (Clemente) C.Agardh		[41,42,63]
*Halymenia harveyana* J.Agardh	*Halymenia floresii* subsp. *harveyana* (J.Agardh) Womersley & Lewis	[41,42,63]
*Halymenia maculata* J.Agardh		[14,27,38,41,42,64,78]
*Halymenia malaysian*a P.-L.Tan, P.-E.Lim, S.-M.Lin & S.-M.Phang		[50]
** *Phyllymenia* J.Agardh**		
*Phyllymenia huangiae* (Showe M.Lin & H.-Y.Liang) Showe M.Lin, Rodríguez-Prieto, De Clerck & Guiry	*Grateloupia huangiae* Showe M.Lin & H.-Y.Liang	[82]
*Phyllymenia taiwanensis* (Showe M.Lin & H.-Y.Liang) Showe M.Lin, Rodríguez-Prieto, De Clerck & Guiry	*Grateloupia taiwanensis* Showe M.Lin & H.Y.Liang	[56]
** *Prionitis* J.Agardh**		
*Prionitis vietnamensis* Pham-Hoàng Hô		[41,42,63]
** Hildenbrandiales**		
** Hildenbrandiaceae**		
** *Actinotrichia* Decaisne**		
*Actinotrichia fragilis* (Forsskål) Børgesen		[14,27,38,41,42,67]
** Nemaliales**		
** Galaxauraceae**		
** *Dichotomaria* Lamarck**		
*Dichotomaria marginata* (J.Ellis & Solander) Lamarck		[41,42,63]
*Dichotomaria obtusata* (J.Ellis & Solander) Lamarck		[38,41,42]
*Dichotomaria papillata* (Kjellman) Kurihara & Masuda		[41,42,63]
** *Galaxaura* J.V.Lamouroux**		
*Galaxaura divaricata* (Linnaeus) Huisman & R.A.Townsend		[38,41,42]
*Galaxaura filamentosa* R.C.Y.Chou		[27,41,42,64,68,72]
*Galaxaura rugosa* (J.Ellis & Solander) J.V.Lamouroux		[38,41,42]
** *Hildenbrandia* Nardo**		
*Hildenbrandia rubra* (Sommerfelt) Meneghini		[38,41,42,64]
** *Tricleocarpa* Huisman & Borowitzka**		
*Tricleocarpa cylindrica* (J.Ellis & Solander) Huisman & Borowitzka	*Galaxaura fastigiata* Decaisne	[38,41,42,64,67,83]
*Tricleocarpa fragilis* (Linnaeus) Huisman & R.A.Townsend		[38,41,42,64]
** Liagoraceae**		
** *Akalaphycus* Huisman, I.A.Abbott & A.R.Sherwood**		
*Akalaphycus setchelliae* (Yamada) Huisman, I.A.Abbott & A.R.Sherwood		[38,41,42]
** *Dermonema* Harvey ex Heydrich**		
*Dermonema pulvinatum* (Grunow) Fan		[27,38,41,42]
*Dermonema virens* (J.Agardh) Pedroche & Ávila Ortíz		[38,41,42]
*Dermonema zinovae* Nguyen Huu Dinh		[41,42,63]
** *Ganonema* K.-C.Fan & Y.-C.Wang**		
*Ganonema farinosum* (J.V.Lamouroux) K.-C.Fan & Y.-C.Wang		[38,41,42,64,67]
*Ganonema pinnatum* (Harvey) Huisman		[41,42,63]
** *Helminthocladia* J.Agardh**		
*Helminthocladia australis* Harvey		[41,42,63]
** *Hommersandiophycus* S.-M.Lin & J.M.Huisman**		
*Hommersandiophycus samaensis* (C.K.Tseng) S.-M.Lin & Huisman	*Ganonema samaense* (C.K. Tseng) Huisman	[38,41,42]
** *Izziella* Doty**		
*Izziella orientalis* (J.Agardh) Huisman & Schils		[38,42]
** *Liagora* J.V.Lamouroux**		
*Liagora ceranoides* J.V.Lamouroux		[14,27,38,41,42]
*Liagora filiformis* K.C.Fan & W.H.Li		[41,42,63]
*Liagora hawaiiana* Butters		[41,42,63]
** *Neoizziella* S.-M.Lin, S.-Y.Yang & Huisman**		
*Neoizziella divaricata* (C.K.Tseng) S.-M.Lin, S.-Y.Yang & Huisman		[38,41,42]
** *Otohimella* Mas.Suzuki**		
*Otohimella japonica* (Yamada) Mas.Suzuki, T.Segawa, Hi.Mori & H.Nozaki	*Liagora japonica* Yamada	[41,42,63]
** *Titanophycus* Huisman, G.W.Saunders & A.R.Sherwood**		
*Titanophycus validus* (Harvey) Huisman, G.W.Saunders & A.R.Sherwood		[41,42,63]
** Scinaiaceae**		
** Scinaia Bivona-Bernardi**		
*Scinaia boergesenii* C.K.Tseng		[41,42,63]
** Yamadaellaceae**		
** *Platoma* Schousboe ex F.Schmitz**		
*Platoma cyclocolpum* (Montagne) F.Schmitz		[41,42,63]
** *Yamadaella* I.A.Abbott**		
*Yamadaella caenomyce* (Decaisne) I.A.Abbott	*Liagora caeomyce* Decaisne	[27,38,41,42]
** Nemastomatales**		
** Schizymeniaceae**		
** *Titanophora* (J.Agardh) Feldmann**		
*Titanophora pikeana* (Dickie) Feldmann		[41,42,63]
*Titanophora weberae* Børgesen	*Titanophora pulchra* E.Y. Dawson	[38,41,42,67]
** Peyssonneliales**		
** Peyssonneliaceae**		
** *Agissea* Pestana, Lyra, Cassano & J.M.C Nunes**		
*Agissea inamoena* (Pilger) Pestana, Lyra, Cassano & J.M.C. Nunes		[27,38,41,42]
*Agissea orientalis* (Weber Bosse) Pestana, Lyra, Cassano & J.M.C Nunes	*Peyssonnelia rubra* f. *orientalis* Weber Bosse	[38,41,42]
** *Peyssonnelia* Decaisne**		
*Peyssonnelia boergesenii* Weber Bosse		[41]
*Peyssonnelia caulifera* Okamura		[41,42,63]
*Peyssonnelia conchicola* Piccone & Grunow		[38,41,42,64,67]
*Peyssonnelia rubra* (Greville) J.Agardh		[14,38,41,42]
** *Ramicrusta* Zhang Derui & Zhou Jinghua**		
*Ramicrusta calcea* (Heydrich) K.R.Dixon & G.W.Saunders	*Peyssonnelia calcea* Heydrich	[14,38,41,42]
** *Sonderophycus* Denizot**		
*Sonderophycus capensis* (Montagne) M.J.Wynne	*Peyssonnelia gunniana* J. Agardh	[41,42,63]
** Plocamiales**		
** Plocamiaceae**		
** *Plocamium* J.V.Lamouroux**		
*Plocamium ovicorne* Okamura		[41]
** Rhodogorgonales**		
** Rhodogorgonaceae**		
** *Rhodogorgon* J.N.Norris & K.E.Bucher**		
*Rhodogorgon ramosissima* J.N.Norris & Bucher	*Rhodogorgon carriebowensis* J.N. Norris & Bucher	[27,38,41,42]
** Rhodymeniales**		
** Champiaceae**		
** *Champia* Desvaux**		
*Champia parvula* (C.Agardh) Harvey		[27,41,42,64,67,68,72]
*Champia salicornioides* Harvey		[41,42,63]
*Champia vieillardii* Kützing		[14,27,41]
** *Coelothrix* Børgesen**		
*Coelothrix irregularis* (Harvey) Børgesen		[41,42,63]
** Faucheaceae**		
** *Gloiocladia* J.Agardh**		
*Gloiocladia japonica* (Okamura) Yoshida		[41]
** Hymenocladiaceae**		
** *Asteromenia* Huisman & A.J.K.Millar**		
*Asteromenia anastomosans* (Weber Bosse) G.W.Saunders, C.E.Lane, C.W.Schneider & Kraft		[38,41,42]
*Asteromenia peltata* (W.R.Taylor) Huisman & A.J.K.Millar		[41,42,63]
** Lomentariaceae**		
** *Ceratodictyon* Zanardini**		
*Ceratodictyon intricatum* (C.Agardh) R.E.Norris		[38,41,42,64]
*Ceratodictyon repens* (Kützing) R.E.Norris	*Gelidiopsis repens* (Kützing) Weber Bosse	[41,42,63]
*Ceratodictyon scoparium* (Montagne & Millardet) R.E.Norris		[38,41,42,67]
*Ceratodictyon spongiosum* Zanardini		[14,27,41,42,67]
*Ceratodictyon variabile* (J.Agardh) R.E.Norris		[38,41,42]
** *Yendoa* C.C.Santos, Lyra & J.M.C.Nunes**		
*Yendoa hakodatensis* (Yendo) C.C.Santos, Lyra & J.M.C.Nunes	*Lomentaria hakodatensis* Yendo	[27,38,41,42]
** Rhodymeniaceae**		
** *Botryocladia* (J.Agardh) Pfeiffer**		
*Botryocladia leptopoda* (J.Agardh) Kylin		[41,42,63]
*Botryocladia skottsbergii* (Børgesen) Levring		[41,42,63]
** *Halichrysis* (J.Agardh) F.Schmitz**		
*Halichrysis micans* (Hauptfleisch) P.Huvé & H.Huvé		[41,42,63]
** *Rhodymenia* Greville**		
*Rhodymenia coacta* Okamura & Segawa		[41,42,63]
*Rhodymenia intricata* (Okamura) Okamura		[41,42,63]
*Rhodymenia liniformis* Okamura		[41,42,63]
** Stylonematales**		
** Stylonemataceae**		
** *Bangiopsis* F.Schmitz**		
*Bangiopsis dumontioides* (P.Crouan & H.Crouan) V.Krishnmurthy		[38,41,42]
** *Chroodactylon* Hansgirg**		
*Chroodactylon ornatum* (C.Agardh) Basson		[27,38,41,42]
** *Stylonema* Reinsch**		
*Stylonema alsidii* (Zanardini) K.M.Drew	*Goniotrichum alsidii* (Zanardini) M. Howe	[27,41,42,64]
**OCHROPHYTA**		
** Dictyotales**		
** Dictyotaceae**		
** *Canistrocarpus* De Paula & De Clerck**		
*Canistrocarpus cervicornis* (Kützing) De Paula & De Clerck		[38,41,42,64,67,68]
*Canistrocarpus crispatus* (J.V.Lamouroux) De Paula & De Clerck		[38,41,42]
** *Dictyopteris* J.V.Lamouroux**		
*Dictyopteris delicatula* J.V.Lamouroux		[41,42,63]
*Dictyopteris plagiogramma* (Montagne) Vickers		[41,42,63]
*Dictyopteris polypodioides* (De Candolle) J.V.Lamouroux		[38,41,42]
*Dictyopteris woodwardia* (R.Brown ex Turner) C.Agardh		[38,41,42]
** *Dictyota* J.V.Lamouroux**		
*Dictyota adnata* Zanardini		[41,42,63]
*Dictyota bartayresiana* J.V.Lamouroux		[38,41,42,64,67,68,72]
*Dictyota ceylanica* var. *anastomosans* Yamada		[14,38,41,42]
*Dictyota ciliolata* Sonder ex Kützing		[38,41,42]
*Dictyota dichotoma* (Hudson) J.V.Lamouroux		[27,41,42,64]
*Dictyota dichotoma* var. *intricata* (C.Agardh) Greville		[41,42,63]
*Dictyota friabilis* Setchell	*Dictyota ceylanica* var. *rotundata* Weber-van-Bosse	[27,41,42,64]
*Dictyota grossedentata* De Clerck & Coppejans		[41,42,63]
*Dictyota hauckiana* Nizamuddin		[58]
*Dictyota implexa* (Desfontaines) J.V.Lamouroux	*Dictyota divaricata* J.V.Lamouroux	[38,41,42,64]
*Dictyota mertensii* (C.Martius) Kützing		[41,42,63]
*Dictyota pinnatifida* Kützing		[41,42,63]
*Dictyota polyclada* Sonder ex Kützing		[41,42,63]
*Dictyota spinulosa* Hooker f. & Arnott		[38,41,42,64,67]
** *Distromium* Levring**		
*Distromium decumbens* (Okamura) Levring		[41,42,63]
** *Lobophora* J.Agardh**		
*Lobophora obscura* (Dickie) C.W.Vieira, De Clerck & Payri		[59]
*Lobophora papenfussii* (W.R.Taylor) Farghaly		[47]
*Lobophora tsengii* D.Tien & Z.Sun		[60]
*Lobophora variegata* (J.V.Lamouroux) Womersley ex E.C.Oliveira		[14,38,41,42]
** *Padina* Adanson**		
*Padina antillarum* (Kützing) Piccone		[38,41,42]
*Padina arborescens* Holmes		[41,42,63]
*Padina australis* Hauck		[14,27,38,41,42]
*Padina australis* var. *cuneata* Tak.Tanaka & K.Nozawa		[41,42,63]
*Padina boryana* Thivy		[41,42,72]
*Padina gymnospora* (Kützing) Sonder		[41,42,68,72]
*Padina japonica* Yamada		[41,42,63]
*Padina minor* Yamada		[41,42,63]
*Padina tetrastromatica* Hauck		[41,42,63]
** *Spatoglossum* Kützing**		
*Spatoglossum schroederi* (C.Agardh) Kützing		[41,42,63]
*Spatoglossum stipitatum* (Tanaka & K.Nozawa) Bittner et al.		[38,41,42]
*Spatoglossum vietnamense* Pham-Hoàng Hô		[14,38,41,42]
** *Stypopodium* Kützing**		
*Stypopodium zonale* (J.V.Lamouroux) Papenfuss		[41,42,63]
** Ectocarpales**		
** Acinetosporaceae**		
** *Feldmannia* Hamel**		
*Feldmannia filifera* (Børgesen) Pham-Hoàng Hô		[41,42,63]
*Feldmannia indica* (Sonder) Womersley & A.Bailey	*Hincksia indica* (Sonder) J.Tanaka	[38,41,42]
*Feldmannia irregularis* (Kützing) Hamel		[14,38,41,42]
*Feldmannia mitchelliae* (Harvey) H.-S.Kim	*Hincksia mitchelliae* (Harvey) P.C.Silva	[27,41,42,64]
** *Herponema* J.Agardh**		
*Herponema zeylanicum* (Børgesen) Krishnamurthy & Baluswami		[41,42,63]
** *Pylaiella* Bory de Saint-Vincent**		
*Pylaiella littoralis* (Linnaeus) Kjellman		[41,42,63]
** Chordariaceae**		
** *Acrothrix* Kylin**		
*Acrothrix pacifica* Okamura & Yamada		[41,42,63]
** *Chilionema* Sauvageau**		
*Chilionema ocellatum* (Kützing) Kornmann		[14,38,41,42]
** *Hecatonema* Sauvageau**		
*Hecatonema enhali* (Børgesen) M.S.Balakrishnan & V.N.Kinkar		[41,42,63]
** *Kuetzingiella* Kornmann**		
*Kuetzingiella elachistiformis* (Heydrich) M.Balakrishnan & Kinkar		[27,38,41,42,67]
** *Myrionema* Greville**		
*Myrionema strangulans* Greville		[41,42,63]
** *Nemacystus* Derbès & Solier**		
*Nemacystus decipiens* (Suringar) Kuckuck		[41,42,63]
** Ectocarpaceae**		
** *Ectocarpus* Lyngbye**		
*Ectocarpus siliculosus* (Dillwyn) Lyngbye		[41]
*Ectocarpus siliculosus* f. *sporangioramosus* A.D.Zinova & Nguyen Huu Dinh		[41,42,63]
*Ectocarpus siliculosus* var. *dasycarpus* (Kuckuck) Gallardo		[41,42,63]
*Ectocarpus vungtauensis* Pham-Hoàng Hô		[41,42,63]
** Scytosiphonaceae**		
** *Chnoospora* J.Agardh**		
*Chnoospora minima* (Hering) Papenfuss		[38,41,42,67]
** *Colpomenia* (Endlicher) Derbès & Solier**		
*Colpomenia sinuosa* (Mertens ex Roth) Derbès & Solier		[41,42,68,72]
** *Dactylosiphon* Santiañez, K.M.Lee, S.M.Boo & Kogame**		
*Dactylosiphon bullosus* (D.A.Saunders) Santiañez, K.M.Lee, S.M.Boo & Kogame		[41,42,63]
** *Hydroclathrus* Bory**		
*Hydroclathrus clathratus* (C.Agardh) M.Howe		[38,41,42,67]
*Hydroclathrus tenuis* C.K.Tseng & Lu Baroen		[38,41,42]
** *Petalonia* Derbès & Solier**		
*Petalonia fascia* (O.F.Müller) Kuntze		[41,42,63]
** *Pseudochnoospora* Santiañez, G.Y.Cho & Kogame**		
*Pseudochnoospora implexa* (J.Agardh) Santiañez, G.Y.Cho & Kogame	*Chnoospora implexa* J.Agardh	[14,27,38,41,42,67]
** *Rosenvingea* Børgesen**		
*Rosenvingea endiviifolia* (Martius) M.J.Wynne		[27,41,42,64,68,72,84]
*Rosenvingea fastigiata* (Zanardini) Børgesen		[38,41,42]
*Rosenvingea nhatrangensis* E.Y.Dawson		[38,41,42,67]
*Rosenvingea orientalis* (J.Agardh) Børgesen		[14,38,41,42,84]
** *Scytosiphon* C.Agardh**		
*Scytosiphon lomentaria* (Lyngbye) Link		[41,42,63]
** Fucales**		
** Sargassaceae**		
** *Hormophysa* Kützing**		
*Hormophysa cuneiformi*s (J.F.Gmelin) P.C.Silva		[41,42,63]
** *Sargassum* C.Agardh**		
*Sargassum aemulum* var. *carpophylloides* Grunow		[41,42,63]
*Sargassum aemulum* var. *jouanii* Grunow		[41,42,63]
*Sargassum angustifolium* C.Agardh		[42,85]
*Sargassum aquifolium* (Turner) C.Agardh		[41,42,85]
*Sargassum armatum* J.Agardh		[41,42,63]
*Sargassum assimile* Harvey		[41,42,63]
*Sargassum baccularia* (Mertens) C.Agardh		[41,42,63]
*Sargassum bangmeianum* Nguyen Huu Dinh & Huynh Quang Nang		[38,41,42]
*Sargassum baorenii* Nguyen Huu Dinh & Huynh Quang Nang		[38,41,42]
*Sargassum bicorne* J.Agardh		[20,38,41,42,85]
*Sargassum brevifolium* var. *pergracile* Grunow		[41,42,63]
*Sargassum bulbiferum* Yoshida		[41,42,63]
*Sargassum buui* Nguyen Huu Dinh & Huynh Quang Nang		[38,41,42]
*Sargassum capillare* Kützing		[41,42,63]
*Sargassum carpophyllum* J.Agardh		[20,38,41,42]
*Sargassum carpophyllum* var. *honomense* Nguyen Huu Dinh & Huynh Quang Nang		[41,42,63]
*Sargassum carpophyllum* var. *nhatrangense* (Pham-Hoang Ho) Ajisaka		[41,42,63]
*Sargassum cinereum* J.Agardh		[41,42,63]
*Sargassum confusum* C.Agardh		[41,42,63]
*Sargassum congkinhii* Pham-Hoàng Hô		[20,38,41,42,85]
*Sargassum cornutifructum* Nguyen Huu Dinh & Huynh Quang Nang		[41,42,63]
*Sargassum cotoense* Nguyen Huu Dai		[41,42,63]
*Sargassum cymosum* C.Agardh		[42,85]
*Sargassum denticarpum* Ajisaka		[41,42,63]
*Sargassum distichum* Sonder		[38,41,42]
*Sargassum emarginatum* C.K.Tseng & Lu Baroen		[38,41,42]
*Sargassum feldmannii* Pham-Hoàng Hô		[38,41,42,85]
*Sargassum flavicans* (Mertens) C.Agardh		[38,41,42,85]
*Sargassum glaucescens* J.Agardh		[38,41,42,85]
*Sargassum gracillimum* Reinbold		[41,42,63]
*Sargassum graminifolium* C.Agardh		[41,42,63]
*Sargassum hemiphyllum* (Turner) C.Agardh		[14,38,41,42]
*Sargassum henslowianum* J.Agardh		[38,41,42,85]
*Sargassum henslowianum* var. *bellonae* Grunow		[41,42,63]
*Sargassum herklotsii* Setchell		[41,42,63]
*Sargassum hieui* Nguyen Huu Dinh & Huynh Quang Nang		[38,41,42]
*Sargassum ilicifolioides* Tseng & Lu		[41,42,63]
*Sargassum ilicifolium* (Turner) C.Agardh		[20,38,41,42,85]
*Sargassum incanum* Grunow		[42,85]
*Sargassum kuetzingii* Setchell		[20,38,41,42,85]
*Sargassum longifructum* C.K.Tseng & B.Lu		[38,41,42,85]
*Sargassum mcclurei* f. *duplicatum* A.D.Zinova & Nguyen Huu Dinh		[41,42,63]
*Sargassum mcclurei* Setchell		[20,41,42,64,68,72]
*Sargassum microcystum* J.Agardh		[27,41,42,64,68,72,85]
*Sargassum miyabei* Yendo		[38,41,42,64,67,68]
*Sargassum namoense* Nguyen Huu Dai		[42,85]
*Sargassum natans* (Linnaeus) Gaillon		[38,41,42]
*Sargassum nipponicum* Yendo		[41,42,63]
*Sargassum oligocystum* Montagne		[20,27,38,41,42,67,85]
*Sargassum paniculatum* J.Agardh		[42,85]
*Sargassum parvifolium* (Turner) C.Agardh		[41,42,63]
*Sargassum parvivesiculosum* C.K.Tseng & B.Lu		[41,42,63]
*Sargassum phamhoangii* Nguyen Huu Dai		[41,42,63]
*Sargassum phyllocystum* C.K.Tseng & Lu Baroen		[41,42,63]
*Sargassum piluliferum* (Turner) C.Agardh		[42,86]
*Sargassum piluliferum* var. *serratifolium* Yamada		[42,85]
*Sargassum polycystum* C.Agardh		[27,41,42,64,67,68,72,85]
*Sargassum polyporum* Montagne		[42,85]
*Sargassum quinhonense* Nguyen Huu Dai		[41,42,63]
*Sargassum segii* Yoshida		[41,42,63]
*Sargassum serratum* Nguyen Huu Dai		[38,41,42,85]
*Sargassum siliquosum* J.Agardh		[38,41,42,85]
*Sargassum subtilissimum* C.K.Tseng & B.Lu		[41,42,63]
*Sargassum swartzii* C.Agardh		[38,41,42,67,85]
*Sargassum tenerrimum* J.Agardh		[41,42,63]
*Sargassum tsengii* Nguyen Huu Dinh & Huynh Quang Nang		[38,41,42]
*Sargassum turbinarioides* Grunow		[41,42,63]
*Sargassum vachellianum* Greville		[42,85]
*Sargassum vietnamense* A.D.Zinova & Dinh		[42,85]
*Sargassum virgatum* C.Agardh		[38,41,42,85]
** *Turbinaria* J.V.Lamouroux**		
*Turbinaria conoides* (J.Agardh) Kützing		[41,42,63]
*Turbinaria decurrens* Bory		[41,42,63]
*Turbinaria gracilis* Sonder		[20,38,41,42,85]
*Turbinaria ornata* (Turner) J.Agardh		[41,42,68,72]
*Turbinaria ornata* var. *prolifera* Pham Hoàng Hô		[41,42,63]
*Turbinaria parvifolia* C.K.Tseng & Lu Baroen		[41,42,63]
*Turbinaria turbinata* (Linnaeus) Kuntze		[41,42,63]
** Ishigeales**		
** Petrodermataceae**		
** *Petroderma* Kuckuck**		
*Petroderma vietnamense* Pham-Hoàng Hô		[41,42,63]
** Ralfsiales**		
** Mesosporaceae**		
** *Mesospora* Weber-van Bosse**		
*Mesospora schmidtii* Weber Bosse		[38,41,42]
** Neoralfsiaceae**		
** *Neoralfsia* P.-E.Lim & H.Kawai**		
*Neoralfsia expansa* (J.Agardh) P.-E.Lim & H.Kawai ex Cormaci & G.Furnari		[38,41,42,64]
** Pseudoralfsiaceae**		
** *Pseudoralfsia* Parente, Fletcher & G.W.Saunders**		
*Pseudoralfsia verrucosa* (Areschoug) Parente, Fletcher & G.W.Saunders	*Ralfsia verrucosa* (Areschoug) Areschoug	[41,42,63]
** Ralfsiaceae**		
** *Ralfsia* Berkeley**		
*Ralfsia fungiformis* (Gunnerus) Setchell & N.L.Gardner		[41,42,63]
** Scytothamnales**		
** Asteronemataceae**		
** *Asteronema* Delépine & Asensi**		
*Asteronema breviarticulatum* (J.Agardh) Ouriques & Bouzon		[38,41,42,64]
** Sphacelariales**		
** Sphacelariaceae**		
** *Sphacelaria* Lyngbye**		
*Sphacelaria carolinensis* Trono		[41,42,63]
*Sphacelaria ceylanica* Sauvageau		[41,42,63]
*Sphacelaria novae-hollandiae* Sonder		[14,27,38,41,42]
*Sphacelaria rigidula* Kützing	*Sphacelaria divaricata* Montagne	[27,41,42,64]
*Sphacelaria solitaria* (Pringsheim) Kylin		[41,42,63]
*Sphacelaria tribuloides* Meneghini		[14,27,38,41,42]
**CHLOROPHYTA**		
** Bryopsidales**		
** Bryopsidaceae**		
** *Bryopsis* J.V.Lamouroux**		
*Bryopsis chapmanii* Molinari & Guiry		[87]
*Bryopsis hypnoides* J.V. Lamouroux		[41,42,63]
*Bryopsis indica* A. Gepp & E.S. Gepp		[14,38,41,42,87]
*Bryopsis pennata* J.V. Lamouroux		[27,38,41,42,64,87]
*Bryopsis pennata* var. *secunda* (Harvey) Collins & Hervey		[41,42,63]
*Bryopsis plumosa* (Hudson) C. Agardh		[27,38,41,42,64,87]
** *Trichosolen* Montagne**		
*Trichosolen mucronatus* (Børgesen) W.R. Taylor		[41,42,63]
*Trichosolen parvus* (E.Y. Dawson) W.R. Taylor	*Pseudobryopsis parva* E.Y.Dawson	[20,38,41,42,87]
** Caulerpaceae**		
** *Caulerpa* J.V.Lamouroux**		
*Caulerpa ambigua* Okamura	*Caulerpella ambigua* (Okamura) Prud’homme & Lokhorst	[14,38,41,42,87]
*Caulerpa andamanensis* (W.R.Taylor) Draisma, Prudhomme & Sauvage		[41,42,63]
*Caulerpa ashmeadii* Harvey		[42,87]
*Caulerpa brachypus* Harvey		[41,42,63]
*Caulerpa chemnitzia* (Esper) J.V.Lamouroux	*Caulerpa peltata* J.V.Lamouroux; *Caulerpa racemosa* var. *occidentalis* (J.Agardh) Børgesen; *Caulerpa racemosa* var. *peltata* (J.V.Lamouroux) Eubank	[41]
*Caulerpa chemnitzia* var. *laetevirens* (Montagne) Fernández-García & Riosmena-Rodriguez		[41,42,63]
*Caulerpa corynephora* Montagne		[41,42,63]
*Caulerpa cupressoide*s (Vahl) C. Agardh		[27,38,41,42,67,87]
*Caulerpa cupressoides* var. *flabellata* Børgesen		[41]
*Caulerpa cupressoides* var. *lycopodium* Weber Bosse		[41,42,63]
*Caulerpa cupressoides* var. *mamillosa* (Montagne) Weber Bosse		[41,42,63]
*Caulerpa falcifolia* Harvey & Bailey		[41,42,63]
*Caulerpa fastigiata* Montagne		[14,38,41,42,67,87]
*Caulerpa lamourouxii* (Turner) C.Agardh	*Caulerpa racemosa* var. *lamourouxii* (Turner) Weber Bosse	
*Caulerpa lentillifera* J. Agardh		[38,41,42,87]
*Caulerpa macrodisca* Decaisne	*Caulerpa peltata* var. *macrodisca* (Decaisne) Weber Bosse	[14,38,41,42,87]
*Caulerpa mexicana* f. *vietnamica* Pham H.H.		[41,42,63]
*Caulerpa mexicana* Sonder ex Kützing		[41,42,63]
*Caulerpa microphysa* (Weber Bosse) Feldmann		[38,41,42,64,87]
*Caulerpa minuta* L.N.Hau		[39]
*Caulerpa nummularia* Harvey ex J. Agardh		[38,41,42,64,67,68]
*Caulerpa oligophylla* Montagne		[41,42,63]
*Caulerpa racemosa* (Forsskål) J. Agardh		[14,27,38,41,42,67,87]
*Caulerpa racemosa* f. *vietnamensis* A.D. Zinova & Nguyen H. Dinh		[42,87]
*Caulerpa racemosa* var. *macrophysa* (Sonder ex Kützing) W.R. Taylor	*Caulerpa macrophysa* (Sonder ex Kützing) G.Murray	[14,27,38,41,42,67,87]
*Caulerpa scalpelliformis* (R. Brown ex Turner) C. Agardh		[42,87]
*Caulerpa serrulata* (Forsskål) J. Agardh	*Caulerpa freycinetii* C.Agardh	[14,27,38,41,42,67,68,72]
*Caulerpa serrulata* f. *lata* (Weber Bosse) C.K. Tseng		[41,42,64]
*Caulerpa serrulata* var. *boryana* (J. Agardh) Gilbert		[41,42,63]
*Caulerpa sertularioides* (S.G. Gmelin) M. Howe		[41,42,63]
*Caulerpa sertularioides* f. *longipes* (J. Agardh) Collins		[41]
*Caulerpa taxifolia* (Vahl) C. Agardh		[41,42,63]
*Caulerpa urvilleana* Montagne	*Caulerpa cupressoides* var. *urvilleana* (Montagne) L.M.Hodgson, P.H. Tri, K.Lewmanomont & K.J. McDermid	[41,42,63]
*Caulerpa verticillata* J. Agardh		[14,27,38,41,42,87]
*Caulerpa verticillata* f. *charoides* Weber Bosse		[41,42,63]
*Caulerpa webbiana* Montagne		[41,42,63]
*Caulerpa webbiana* f. *tomentella* (Harvey ex J. Agardh) Weber Bosse		[41,42,63]
*Caulerpa sertularioides* f. *longiseta* (Bory) Svedelius		[41,42,63]
** Codiaceae**		
** *Codium* Stackhouse**		
*Codium adhaerens* C. Agardh		[14,38,41,42,87]
*Codium arabicum* Kützing		[14,38,41,42,67,87]
*Codium cylindricum* Holmes		[41,42,63]
*Codium duthieae* P.C. Silva		[42,87]
*Codium formosanum* Yamada		[41,42,63]
*Codium geppiorum* O.C. Schmidt		[27,38,41,42,64,87]
*Codium intricatum* Okamura		[41,42,63]
*Codium isthmocladum* Vickers		
*Codium mamillosum* Harvey		[38]
*Codium repens* P.L. Crouan & H.M. Crouan		[38,41,42,64,67,87]
*Codium tomentosum* Stackhouse		[41,42,63]
** *Geppella* Børgesen**		
*Geppella prolifera* C.K. Tseng & M.L. Dong		[41,42,63]
** Derbesiaceae**		
** *Derbesia* Solier**		
*Derbesia attenuata* E.Y. Dawson		[14,27,38,41,42,87]
*Derbesia marina* (Lyngbye) Solier		[42,87]
** *Halicystis* Areschoug**		
*Halicystis pyriformis* Levring		[41,42,63]
** Dichotomosiphonaceae**		
** *Avrainvillea* Decaisne**		
*Avrainvillea amadelpha* (Montagne) A. Gepp & E. Gepp		[41,42,63]
*Avrainvillea erecta* (Berkeley) A. Gepp & E. Gepp		[14,38,41,42,67,87]
*Avrainvillea lacerata* Harvey ex J. Agardh		[41,42,63]
*Avrainvillea obscura* (C. Agardh) J. Agardh	*Avrainvillea capituliformis* T.Tanaka	[41,42,63]
** Halimedaceae**		
** *Boodleopsis* A.Gepp & E.S.Gepp**		
*Boodleopsis pusilla* (Collins) W.R.Taylor, A.B.Joly & Bernatowicz		[41,42,63]
** *Chlorodesmis* Harvey & Bailey**		
*Chlorodesmis hildebrandtii* A. Gepp & E. Gepp		[14,38,41,42,87]
** *Halimeda* J.V.Lamouroux**		
*Halimeda cuneata* f. *digitata* E.S. Barton		[14,38,41,42]
*Halimeda cuneata* Hering		[41,42,63]
*Halimeda cylindracea* Decaisne		[41,42,63]
*Halimeda discoidea* Decaisne		[14,27,41,42,67,87]
*Halimeda gracilis* Harvey ex J. Agardh		[38,41,42]
*Halimeda incrassata* (J. Ellis) J.V. Lamouroux		[14,38,41,42,87]
*Halimeda macroloba* Decaisne		[41,42,63]
*Halimeda micronesica* Yamada		[41,42,63]
*Halimeda opuntia* (Linnaeus) J.V. Lamouroux		[14,27,38,41,42,67,68,72,87]
*Halimeda taenicola* W.R. Taylor		
*Halimeda tuna* (J. Ellis et Solander) J.V. Lamouroux		[14,38,41,42,87]
*Halimeda velasquezii* W.R. Taylor		[41,42,63]
*Halimeda xishaensis* C.K. Tseng & M.L. Dong		[41,42,63]
** *Penicillus* Lamarck**		
*Penicillus sibogae* A. Gepp & E. Gepp		[14,38,41,42,87]
** *Pseudochlorodesmis* Børgesen**		
*Pseudochlorodesmis furcellata* (Zanardini) Børgesen		[14,38,41,42,87]
** *Rhipidosiphon* Montagne**		
*Rhipidosiphon javensis* Montagne	*Udotea javensis* (Montagne) A.Gepp & E.S.Gepp	[14,38,41,42,87]
** *Rhipiliopsis* A.Gepp & E.S.Gepp**		
*Rhipiliopsis echinocaulos* (A.B. Cribb) Farghaly		[41,42,63]
** *Tydemania* Weber Bosse**		
*Tydemania expeditionis* Weber Bosse		[41,42,63]
** *Udotea* J.V.Lamouroux**		
*Udotea argentea* Zanardini		[41,42,63]
*Udotea flabellum* (J. Ellis & Solander) M. Howe		[41,42,63]
*Udotea orientalis* A. Gepp & E. Gepp		[41,42,63]
*Udotea velutina* C.K. Tseng & M.L. Dong		[41,42,63]
** Ostreobiaceae**		
** *Ostreobium* Bornet & Flahault**		
*Ostreobium quekettii* Bornet & Flahault		[38,41,42]
** Pseudobryopsidaceae**		
** *Pseudobryopsis* Berthold**		
*Pseudobryopsis hainanensis* C.K. Tseng		[41,42,63]
** Cladophorales**		
** Anadyomenaceae**		
** *Anadyomene* J.V.Lamouroux**		
*Anadyomene plicata* C.Agardh		[14,38,41,42,67,87]
*Anadyomene wright*ii Harvey ex J.E. Gray		[14,27,41,42,67,87]
** *Microdictyon* Decaisne**		
*Microdictyon japonicum* Setchell		[27,38,41,42,67]
*Microdictyon nigrescens* (Yamada) Setchell		[42,87]
*Microdictyon okamurae* Setchell		[38,41,42,64,87]
*Microdictyon vanbosseae* Setchell		[42,87]
** Boodleaceae**		
** *Boodlea* G.Murray & De Toni**		
*Boodlea coacta* (Dickie) G. Murray & De Toni		[41,42,63]
*Boodlea composita* (Harvey) F. Brand		[14,27,38,41,42]
*Boodlea struveoides* M. Howe		[38,41,42,64,87]
** *Cladophoropsis* Børgesen**		
*Cladophoropsis fasciculata* (Kjellman) Wille		[38,41,42,64]
*Cladophoropsis membranacea* (Hofman ex C. Agardh) Børgesen		[14,27,38,41,42,87]
*Cladophoropsis phamhoanghoii* Molinari & Guiry		[14,27,38,41,42,87]
*Cladophoropsis vaucheriiformis* (Areschoug) Papenfuss		[14,27,38,41,42,87]
** *Phyllodictyon* J.E.Gray**		
*Phyllodictyon anastomosans* (Harvey) Kraft & M.J.		[38,41,42,64]
** Cladophoraceae**		
** *Chaetomorpha* Kützing**		
*Chaetomorpha aerea* (Dillwyn) Kützing	*Chaetomorpha crassa* (C.Agardh) Kützing	[14,38,41,42,87]
*Chaetomorpha antennina* (Bory) Kützing		[38,41,42,87]
*Chaetomorpha basiretrorsa* Setchell		[41,42,63]
*Chaetomorpha gracilis* Kützing		[41,42,63]
*Chaetomorpha indica* (Kützing) Kützing		[38,41,42,64,87]
*Chaetomorpha javanica* Kützing		[41,42,63]
*Chaetomorpha linum* (O.F. Müller) Kützing		[41,42,63]
*Chaetomorpha pachynema* (Montagne) Kützing		[42,87]
*Chaetomorpha spiralis* Okamura		[38,41,42,87]
*Chaetomorpha tortuosa* (Dillwyn) Kleen		[42,87]
** *Cladophora* Kützing**		
*Cladophora adhaerens* Harvey		[41,42,63]
*Cladophora albida* (Nees) Kützing		[41,42,63]
*Cladophora aokii* Yamada		[41,42,63]
*Cladophora catenata* (Linnaeus) Kützing		[38,41,42]
*Cladophora coelothrix* Kützing		[41,42,63]
*Cladophora crispula* Vickers		[41,42,63]
*Cladophora flexuosa* (O.F. Müller) Kützing		[38,41,42,64]
*Cladophora glomerata* (Linnaeus) Kützing		[41,42,63]
*Cladophora gracilis* Kützing		[41,42,63]
*Cladophora laetevirens* (Dillwyn) Kützing		[27,38,41,42,64,87]
*Cladophora papenfussii* Pham H.H.		[41,42,63]
*Cladophora patentirame*a (Montagne) Kützing		[41,42,63]
*Cladophora perpusilla* Skottsberg &Levring		[38,41,42,87]
*Cladophora prolifera* (Roth) Kützing		[38,41,42]
*Cladophora rugulosa* G.Martens		[41,42,63]
*Cladophora sericea* (Hudson) Kützing		[41,42,63]
*Cladophora socialis* Kützing		[14,27,38,41,42]
*Cladophora stimpsonii* Harvey		[42,87]
*Cladophora vagabunda* (Linnaeus) C. Hoek	*Cladophora uncinella* Harvey; Cladophora inserta f. ungulata (Brand) Setchell)	[27,38,41,42,67,68]
** *Lychaete* J.Agardh**		
*Lychaete herpestica* (Montagne) M.J.Wynne		[38,42]
*Lychaete pellucida* (Hudson) M.J.Wynne		[42,87]
*Lychaete sakaii* (I.A.Abbott) M.J.Wynne		[41,42,63]
** *Rhizoclonium* Kützing**		
*Rhizoclonium grande* Børgesen		[14,38,41,42,87]
*Rhizoclonium riparium* (Roth) Harvey	*Rhizoclonium implexum* (Dillwyn) Kützing	[38,41,42,64,87]
** Siphonocladaceae**		
** *Boergesenia* J.Feldmann**		
*Boergesenia forbesii* (Harvey) Feldmann		[14,38,41,42,87]
** *Dictyosphaeria* Decaisne**		
*Dictyosphaeria cavernosa* (Forsskål) Børgesen		[14,27,41,42,67,87]
*Dictyosphaeria spinifera* C.K. Tseng & C.F. Chang		[42,87]
*Dictyosphaeria versluysii* Weber Bosse		[14,27,38,41,42]
** Valoniaceae**		
** *Valonia* C.Agardh**		
*Valonia aegagropila* C. Agardh		[14,38,41,42,67,87]
*Valonia fastigiata* Harvey ex J. Agardh		[14,38,41,42,67,87]
*Valonia macrophysa* Kützing		
*Valonia utricularis* (Roth) C. Agardh		[14,38,41,42,67,87]
*Valonia ventricosa* J. Agardh		[41,42,63]
** *Valoniopsis* Børgesen**		
*Valoniopsis pachynema* (G. Martens) Børgesen		[41,42,63]
** Dasycladales**		
** Bornetellaceae**		
** *Bornetella* Munier-Chalmas**		
*Bornetella nitida* Munier-Chalmas ex Sonder		[41,42,63]
*Bornetella oligospora* Solms-Laubach		[41,42,63]
*Bornetella sphaerica* (Zanardini) Solms-Laubach		[14,38,41,42,67,87]
** Dasycladaceae**		
** *Neomeris* J.V.Lamouroux**		
*Neomeris annulata* Dickie		[14,27,41,42,67,87]
*Neomeris bilimbata* J.T. Koster		[38,41,42,64,87]
*Neomeris vanbosseae* M. Howe		[14,27,41,42,67,87]
** Polyphysaceae**		
** *Acetabularia* J.V.Lamouroux**		
*Acetabularia caliculus* J.V.Lamouroux		[14,38,41,42,67,87]
*Acetabularia major* G. Martens		[41,42,63]
** *Parvocaulis* S.Berger, U.Fettweiss, S.Gleissberg, L.B.Liddle, U.Richter, H.Sawitzky & G.C.Zuccarello**		
*Parvocaulis clavatus* (Yamada) S. Berger, U. Fettweiss, S. Gleissberg, L.B. Liddle, U. Richter, H. Sawitzky & Zucca- rello		[38,41,42]
*Parvocaulis exiguus* (Solms-Laubach) S.Berger, Fettweiss, Gleissberg, Liddle, U.Richter, Sawitzky & Zuccarello		[41,42,63]
*Parvocaulis parvulus* (Solms-Laubach) S. Berger, U. Fett- weiss, S. Gleissberg, L.B. Liddle, U. Richter, H. Sawitzky & Zuccarello		[38,41,42]
*Parvocaulis pusillus* (M. Howe) S. Berger, U. Fettweiss, S. Gleissberg, L.B. Liddle, U. Richter, H. Sawitzky & Zucca- rello		[38,41,42]
** Ulotrichales**		
** Gayraliaceae**		
** *Gayralia* K.L.Vinogradova**		
*Gayralia oxysperma* (Kützing) K.L. Vinogradova ex Scagel, R.F., Gabrielson, P.W., Garbary, D.J., Golden, L., Hawkes, M.W., Lindstrom, S.C., Oliveira, J.C. & Widdowson, T.B.		[41,42,63]
Gomontiaceae		
** *Gomontia* Bornet & Flahault**		
*Gomontia arrhiza* Hariot		[41,42,63]
** Monostromataceae**		
** *Monostroma* Thuret**		
*Monostroma nitidum* Wittrock	*Porphyra crispata* Kjellman	[14,38,41,42,87]
** Ulotrichaceae**		
** *Ulothrix* Kützing**		
*Ulothrix flacca* (Dillwyn) Thuret		[42,87]
*Ulothrix subflaccida* Wille		[41,42,63]
** Ulvales**		
** Ulvaceae**		
** *Enteromorpha* Link**		
*Enteromorpha stipitata* var. *catbaenis* A.D. Zinova & Nguyen H. Dinh		[87]
** *Ulva* Linnaeus**		
*Ulva aragoënsis* (Bliding) Maggs		[61]
*Ulva chaetomorphoides* (Børgesen) Hayden, Blomster, Maggs, P.C. Silva, M.J. Stanhope et J.R. Waaland		[38,41,42,64,72]
*Ulva chaugulii* M.G.Kavale & M.A.Kazi		[61]
*Ulva clathrata* (Roth) C. Agardh		[38,41,42,88]
*Ulva compressa* Linnaeus		[38,41,42,64,72]
*Ulva conglobata* Kjellman		[38,41,42,64,72]
*Ulva fenestrata* Postels & Ruprecht		[38,41,42,64,72]
*Ulva flexuosa* subsp. *pilifera* (Kützing) M.J. Wynne		[41,42,63]
*Ulva flexuosa* Wulfen		[38,41,42,64,72]
*Ulva intestinalis* Linnaeus		[38,41,42,64,72]
*Ulva kraftiorum* Huisman		[61]
*Ulva kylinii* (Bliding) Hayden, Blomster, Maggs, P.C. Silva, M.J. Stanhope et J.R. Waaland		[38,41,42,64,72]
*Ulva lactuca* Linnaeus		[38,41,42,64,72]
*Ulva limnetica* K.Ichihara & S.Shimada		[61]
*Ulva meridionalis* R.Horimoto & S.Shimada		[61]
*Ulva ohnoi* M.Hiraoka & S.Shimada		[61]
*Ulva papenfussii* Pham H.H.		[38,41,42,64,72]
*Ulva prolifera* O.F. Müller		[38,41,42,64,72]
*Ulva ralfsii* (Harvey) Le Jolis		[38,41,42,64,72]
*Ulva reticulata* Forsskål		[27,38,41,42,64,87,88]
*Ulva spinulosa* Okamura & Segawa		[42,87]
*Ulva tepida* Y.Masakiyo & S.Shimada		[61]
*Ulva torta* (Mertens) Trevisan		[41,42,63]
*Ulva vietnamensis* L.-A.T.Tran, Leliaert & De Clerck		[61]
** Ulvellaceae**		
** *Ulvella* P.L.Crouan & H.M.Crouan**		
*Ulvella lens* P.L. Crouan et H.M. Crouan		[14,38,41,42,87]
*Ulvella leptochaete* (Huber) R.Nielsen, C.J.O’Kelly & B.Wysor		[41,42,63]
*Ulvella scutata* (Reinke) R.Nielsen, C.J.O’Kelly & B.Wysor		[41,42,63]
*Ulvella viridis* (Reinke) R. Nielsen, C.J. O’Kelly & B. Wysor		[41,42,63]
** Vaucheriales**		
** Vaucheriaceae**		
** *Vaucheria* A.P.de Candolle**		
*Vaucheria piloboloides* Thuret		[41,42,63]
**TRACHEOPHYTA**		
** Alismatales**		
** Cymodoceaceae**		
** *Cymodocea* K.D.König**		
*Cymodocea rotundata* Ascherson & Schweinfurth		[89]
** *Halodule* Endlicher**		
*Halodule pinifolia* (Miki) Hartog		[44]
*Halodule uninervis* (Forsskål) Ascherson		[44]
** *Oceana* Byng & Christenhusz**		
*Oceana serrulata* (R.Brown) Byng & Christenhusz	*Cymodocea serrulata* (R.Brown) Ascherson & Magnus	[89]
** *Syringodium* Kützing**		
*Syringodium isoetifolium* (Ascherson) Dandy		[44]
** *Thalassodendron* Den Hartog**		
*Thalassodendron ciliatum* (Forsskål) Hartog		[89]
** Hydrocharitaceae**		
** *Enhalus* L.C. Richard**		
*Enhalus acoroides* (Linnaeus f.) Royle		[89]
** *Halophila* Thouars**		
*Halophila beccarii* Ascherson		[44]
*Halophila decipiens* Ostenfeld		[44]
*Halophila major* (Zollinger) Miquel		[44]
*Halophila minor* (Zollinger) Hartog		[90]
*Halophila ovalis* (R.Brown) Hooker f.		[44,90]
** *Thalassia* Banks ex König**		
*Thalassia hemprichii* (Ehrenberg) Ascherson		[89]
** Ruppiaceae**		
** *Ruppia* Linnaeus**		
*Ruppia maritima* Linnaeus		[91]
** Zosteraceae**		
** Zostera Linnaeus**		
*Zostera japonica* Ascherson & Graebner		[92]

**Table 3:** Species diversity of marine floral groups in countries bordering the South China Sea (East Vietnam Sea). Data based on AlgaeBase [46]. * References for seagrasses (Alismatales).

Countries	Cyanobacteria	Rhodophyta	Ochrophyta	Chlorophyta	Alima-Stales	Ref. *
China	114	495	301	205	22	[113]
Indonesia	11	483	165	232	13	[114]
Malaysia	2	85	45	39	16	[115]
Philippines	6	597	183	244	18	[116]
Singapore	15	114	92	97	10	[117]
Thailand	0	76	40	77	12	[118]
Vietnam	51	438	171	202	15	[119]

**Table 4:** Endemic marine algae species from Vietnam.

No.	Phylum	Genus	Name Species
1	Rhodophyta	*Acrochaetium* Nägeli	*Acrochaetium gracile* var. *vietnamense* Pham-Hoàng Hộ
2			*Acrochaetium phuquocense* Pham-Hoàng Hộ
3			*Acrochaetium pseudoerectum* Pham-Hoàng Hộ
4		*Ahnfeltiopsis* P.C.Silva & DeCew	*Ahnfeltiopsis quinhonensis* (Pham-Hoàng Hộ) Masuda
5		*Bangia* Lyngbye	*Bangia tanakae* Pham-Hoàng Hộ
6		*Caloglossa* (Harvey) G.Martens	*Caloglossa saigonensis* Tanaka & Pham-Hoàng Hộ
7		*Ceramium* Roth	*Ceramium vietnamense* Pham-Hoàng Hộ
8		*Ectocarpus* Lyngbye	*Ectocarpus siliculosus* f. *sporangioramosus* A.D.Zinova & Nguyen Huu Dinh
9			*Ectocarpus vungtauensis* Pham-Hoàng Hộ
10		*Erythrotrichia* Areschoug	*Erythrotrichia parietalis* var. *majuscula* T.Tanaka & Pham-Hoàng Hộ
11		*Gelidium* J.V.Lamouroux	*Gelidium vietnamense* Pham-Hoàng Hộ
12		*Gracilariopsis* E.Y.Dawson	*Gracilariopsis nganii* Pham-Hoàng Hộ
13			*Gracilariopsis nhatrangensis* Nhu Hau Le & S.-M.Lin
14			*Gracilariopsis phanthietensis* Pham-Hoàng Hộ
15		*Herposiphonia* Nägeli	*Herposiphonia vietnamica* Pham-Hoàng Hộ
16		*Meristotheca* J.Agardh	*Meristotheca lysonensis* X.-V.Nguyen, X.-T.Nguyen, Kittle & McDermid
17		*Porphyra* C.Agardh	*Porphyra tanakae* Pham-Hoàng Hộ
18		*Prionitis* J.Agardh	*Prionitis vietnamensis* Pham-Hoàng Hộ
19		*Pyropia* J.Agardh	*Pyropia vietnamensis* (Tak.Tanaka & P.H.Hô) J.E.Sutherland & Monotilla
20		*Rodriguezella* F.Schmitz	*Rodriguezella hongngai* Pham-Hoàng Hộ
21	Ochrophyta	*Lobophora* J.Agardh	*Lobophora tsengii* D.Tien & Z.Sun
22		*Petroderma* Kuckuck	*Petroderma vietnamensis* Pham-Hoàng Hộ
23		*Sargassum* C.Agardh	*Sargassum bangmeianum* Nguyen Huu Dinh & Huynh Quang Nang
24			*Sargassum baorenii* Nguyen Huu Dinh & Huynh Quang Nang
25			*Sargassum buui* Nguyen Huu Dinh & Huynh Quang Nang
26			*Sargassum carpophyllum* var. *honomense* Nguyen Huu Dinh & Huynh Quang Nang
27			*Sargassum carpophyllum* var. *nhatrangense* (Pham-Hoàng Hộ) Ajisaka
28			*Sargassum congkinhii* Pham-Hoàng Hộ
29			*Sargassum cornutifructum* Nguyen Huu Dinh & Huynh Quang Nang
30			*Sargassum cotoense* Nguyen Huu Dai
31			*Sargassum hieui* Nguyen Huu Dinh & Huynh Quang Nang
32			*Sargassum mcclurei* f. *duplicatum* A.D.Zinova & Nguyen Huu Dinh
33			*Sargassum namoense* Nguyen Huu Dai
34			*Sargassum phamhoangii* Nguyen Huu Dai
35			*Sargassum quinhonense* Nguyen Huu Dai
36			*Sargassum serratum* Nguyen Huu Dai
37			*Sargassum tsengii* Nguyen Huu Dinh & Huynh Quang Nang
38			*Sargassum vietnamense* A.D.Zinova & Dinh
39		*Spatoglossum* Kützing	*Spatoglossum vietnamense* Pham-Hoàng Hộ
40		*Turbinaria* J.V.Lamouroux	*Turbinaria ornata* var. *prolifera* Pham-Hoàng Hộ
41	Chlorophyta	*Caulerpa* J.V.Lamouroux	*Caulerpa mexicana* f. *vietnamica* Pham-Hoàng Hộ
42			*Caulerpa minuta* L.N.Hau
43			*Caulerpa racemosa* f. *vietnamensis* A.D. Zinova & Nguyen H. Dinh
44		*Cladophoropsis* Børgesen	*Cladophoropsis phamhoanghoii* Molinari & Guiry
45		*Ulva* Linnaeus	*Ulva vietnamensis* L.-A.T.Tran, Leliaert & De Clecrck

**Table 5:** Checklist MPAs in Vietnam.

No.	MPA Name	Province	Regions	Area (ha)	Typical Species
1	Co To-Dao Tran	Quang Ninh	Southeast	18,400	Coral, seaweed, seagrass
2	Bai Tu Long	Quang Ninh	Southeast	96,500	Coral, seagrass
3	Cat Ba	Hai Phong	Red River Delta	10,900	Coral, seaweed, seagrass
4	Bach Long Vi	Hai Phong	Red River Delta	27,000	Coral, abalone
5	Con Co	Quang Tri	North Central Coast	2140	Red coral, seagrass
6	Cu Lao Cham	Quang Nam	South Central Coast	6716	Coral, seaweed, seagrass
7	Ly Son	Quang Ngai	South Central Coast	7925	Coral, seagrass
8	Nha Trang Bay	Khanh Hoa	South Central Coast	12,000	Coral, seagrass
9	Nui Chua	Ninh Thuan	South Central Coast	7352	Coral, sea turtle
10	Hon Cau	Binh Thuan	South Central Coast	12,390	Coral, seagrass, rock crab, shrimp
11	Con Dao	Ba Ria–Vung Tau	South Central Coast	23,000	Dugong, sea turtle, coral
12	Phu Quoc	Kien Giang	Mekong River Delta	18,700	Coral, seagrass
Total area (ha)				243,023	-

**Table 6:** Nature reserves and national parks in Vietnam.

Nature Reserve	Province	Regions	Area (ha)
Dong Rui Wetland Conservation Area	Quang Ninh	Southeast	15,750
Cat Ba Biosphere Reserve	Hai Phong	Red River Delta	26,588
Thai Thuy Wetland Conservation Area	Thai Binh	Red River Delta	6560
Red River Delta Biosphere Reserve	Thai Binh-Nam Dinh-Ninh Binh	Red River Delta	105,558
Tam Giang–Cau Hai Wetland Nature Reserve	Thua Thien Hue	North Central Coast	2071
Son Tra Nature Reserve	Da Nang	North Central Coast	4400
Nui Chua National Park	Ninh Thuan	South Central Coast	29,865
Binh Chau–Phuoc Buu Nature Reserve	Ba Ria—Vung Tau	Southeast Coast	10,537
Can Gio Biosphere Reserve	Ho Chi Minh	Southeast Coast	75,740
Mui Ca Mau National Park	Ca Mau	Mekong River Delta	41,862

**Table 7:** Biosecurity components adopted in the national seaweed policies and regulations in the main *Gracilaria* and *Kappaphycus alvarezii* production.

Biosecurity Component	Policy/Regulation	Description
Established aquatic animal/plant regulation	Regulation No. 434/2021	National plan for prevention and control of some dangerous diseases on aquaculture
Disease surveillance system	Plant Protection and Quarantine Law No. 41/2013	Law on activities of preventing and combating organisms harmful to plants, phytosanitary, management of pesticides
National regulation of live seaweed movement	Fisheries Law No. 18/2017	Law on fisheries activities, state management of fisheries
National statutory contingency plan	Regulation No. 1664/2021	Law on fisheries activities, state management of fisheries
National standards of seaweed cultivation, production process, food safety	- TCN 108:1998; - TCN 155:2000; - TCVN 10371:2014	- Ministry standard for *Gracilaria asiatica* planting material; - Ministry standard for technical process of cultivation of *Gracilaria asiatica* to produce 2 tons dry seaweed/ha/year; - Technical requirements for *Kappaphycus alvarezii.*

## Data Availability

Not applicable.
